# Target Proteins in the Dorsal Hippocampal Formation Sustain the Memory-Enhancing and Neuroprotective Effects of *Ginkgo biloba*

**DOI:** 10.3389/fphar.2018.01533

**Published:** 2019-01-07

**Authors:** Renan Barretta Gaiardo, Thiago Ferreira Abreu, Alexandre Keiji Tashima, Monica Marques Telles, Suzete Maria Cerutti

**Affiliations:** ^1^Departamento de Ciências Biológicas, Laboratório de Farmacologia Celular e Comportamental, Universidade Federal de São Paulo, Diadema, Brazil; ^2^Departamento de Bioquímica, Escola Paulista de Medicina, Universidade Federal de São Paulo, São Paulo, Brazil; ^3^Departamento de Ciências Biológicas, Laboratório de Fisiologia Metabólica, Universidade Federal de São Paulo, Diadema, Brazil

**Keywords:** conditioned suppression, proteomic, *Ginkgo biloba*, long-term memory, protein–protein interaction

## Abstract

We have previously shown that standardized extracts of *Ginkgo biloba* (EGb) modulate fear memory formation, which is associated with CREB-1 (mRNA and protein) upregulation in the dorsal hippocampal formation (dHF), in a dose-dependent manner. Here, we employed proteomic analysis to investigate EGb effects on different protein expression patterns in the dHF, which might be involved in the regulation of CREB activity and the synaptic plasticity required for long-term memory (LTM) formation. Adult male Wistar rats were randomly assigned to four groups (*n* = 6/group) and were submitted to conditioned lick suppression 30 min after vehicle (12% Tween 80) or EGb (0.25, 0.50, and 1.00 g⋅kg^−1^) administration (p.o). All rats underwent a retention test session 48 h after conditioning. Twenty-four hours after the test session, the rats were euthanized *via* decapitation, and dHF samples were removed for proteome analysis using two-dimensional polyacrylamide gel electrophoresis, followed by peptide mass fingerprinting. In agreement with our previous data, no differences in the suppression ratios (SRs) were identified among the groups during first trial of CS (conditioned stimulus) presentation (*P* > 0.05). Acute treatment with 0.25 g⋅kg^−1^ EGb significantly resulted in retention of original memory, without prevent acquisition of extinction within-session. In addition, our results showed, for the first time, that 32 proteins were affected in the dHF following treatment with 0.25, 0.50, and 1.00 g⋅kg^−1^ doses of EGb, which upregulated seven, 19, and five proteins, respectively. Additionally, EGb downregulated two proteins at each dose. These proteins are correlated with remodeling of the cytoskeleton; the stability, size, and shape of dendritic spines; myelin sheath formation; and composition proteins of structures found in the membrane of the somatodendritic and axonal compartments. Our findings suggested that EGb modulates conditioned suppression LTM through differential protein expression profiles, which may be a target for cognitive enhancers and for the prevention or treatment of neurocognitive impairments.

## Introduction

The ability to extract meaning from sensory input, i.e., acquiring new knowledge about the world, and storing this information as past experiences for subsequent retrieval is part of the normal development of living beings and has been well studied in invertebrates animals (Carew and Sahley, 1986; Kandel et al., 2014). These events are largely coordinated and are crucial to memory formation, being characterized by specific cellular and/or molecular modifications that might last from seconds or minutes to hours. If these modifications persist over time, they guarantee that long-lasting changes, which are essential to long-term memory (LTM) formation, will occur (Stork, 1999; Won and Silva, 2008; Mayford et al., 2012). In vertebrates, LTM seems to occur in parallel on the day of training within the specific neural circuitry for each memory type and may involve the hippocampus, prefrontal cortex, and amygdaloid complex (Izquierdo et al., 1997; Cammarota et al., 2008).

The early phase of LTM occurs as a result of transient modifications to the activity of already existing proteins that are present on the membrane or in the cytosol of a neural cell, which act by regulating ionic conductivity as NMDA receptors or by phosphorylation of proteins as alpha calcium/calmodulin-dependent kinase II and the mitogen-activated protein kinases family as ERK1/2 (Fendt and Fanselow, 1999; Giese and Mizuno, 2013). The maintenance of these signals, in parallel with other downstream activation targets, is necessary for long-lasting neuronal plasticity and cellular consolidation of memory, a protein synthesis-dependent stage of memory formation that may last for hours, weeks, or months.

Subsequent evidence has demonstrated that *de novo* proteins have multiple functions in cells and are essential for dendritic spicule growth, functional synaptic area increase, and synaptic cleft narrowing, which are involved in new synaptic formation and/or in the strength of existing synapses over time. Furthermore, changes may occur through underutilized synapses that weaken or eliminate some inputs. Both mechanisms provide a cellular substrate for the formation of learned associations (McGaugh, 1966; Frey et al., 1993; Hotulainen and Hoogenraad, 2010).

In addition to the neuronal adaptations, the cross-talk between neurons and glial cells is crucial to the maintenance of the appropriate environment for neural functioning, electrochemical homeostasis, transmitter release, memory formation, or the effects of drug therapy (Lynch, 2002; Barco et al., 2003; Cerutti et al., 2009). Hence, beyond the synaptic and extrasynaptic changes that underlie memory formation, alterations in the intrinsic cell functions, such as redox balance regulation and hypoxia tolerance, may guarantee better conditions and, consequently, better cell functioning, which promote memory maintenance (Kandel, 2001).

In recent decades, popular and institutional interest in the use of herbal medicines as a therapeutic resource in the prevention, promotion, and recovery of health has grown in many countries. Currently, the standardized extract of *Ginkgo biloba* (EGb) is the plant product that has been most frequently indicated for the treatment and prevention of the loss of function related to ageing of the nervous system, in particular for treatment of diseases associated with cognitive and memory dysfunctions (Itil et al., 1996; DeFeudis, 2002; Ahlemeyer and Krieglstein, 2003b).

Studies from our laboratory have characterized the protective and preventive effects of EGb treatment on the dorsal hippocampal formation (dHF) of middle-aged rats, which is associated with short-term memory (Ribeiro et al., 2016), or of adult rats during LTM enhancement (Oliveira et al., 2009, 2013; Zamberlam et al., 2016). Our group showed that upregulation of CREB-1 in the dHF is essential for conditioned suppression memory and that short or long-term treatment with EGb upregulated the expression of CREB-1 (mRNA and protein) and of glial fibrillary acidic protein in the dHF in a dose-dependent manner (Oliveira et al., 2009, 2013). Conversely, EGb treatment downregulated the product of the gene expression (mRNA and protein) of growth associated protein 43 (also called neuromodulin), a neural-specific protein kinase C substrate, in the dHF of rats subjected to the suppression of licking response test in parallel with cellular and molecular changes in the prefrontal cortex and the amygdaloid complex. Recently, we suggested that treatment with EGb prior to conditioned suppression modulates molecular mechanisms that are associated with acquisition or consolidation, resulting in the retention of fear memory over time (Zamberlam et al., 2016). These pieces of evidence were recently corroborated since we found that the effects of EGb treatment on the modulation of fear memory retrieval over time were correlated with differential expression patterns of serotoninergic, GABAergic, and glutamatergic receptors in the dHF (Zamberlam et al., unpublished). Altogether, these findings established the proprieties of EGb on memory and raised questions about the role of the dHF as a key structure in the acquisition of conditioned lick suppression. However, intrahippocampal downstream proteins that may be involved in the regulation of CREB activity, as well as in the synaptic plasticity required for LTM formation, have not been characterized.

To address these questions, we employed quantitative proteomic analysis to investigate the effects of treatment with EGb before conditioning on differential protein expression.

## Materials and Methods

### Animals

Twenty-four male adult *Wistar* rats, 12 weeks old (250–300 g) were obtained from the Center for the Development of Experimental Medicine and Biology, Universidade Federal de São Paulo (São Paulo, Brazil). All animals were experimentally naive and were housed three per cage with food and water freely available. They were kept under a controlled temperature of 21 ± 2°C, a relative humidity of 53 ± 2%, and a 12:12 h light cycle with lights on at 06:00 hours throughout the experimental period. The experiments were performed in the light phase of the cycle. All procedures were approved by the Ethics Committee on Animal Use of the Federal University of Sao Paulo (CEUA 0043/12) and in accordance with the rules issued by the National Council for the Control of Animal Experimentation (CONCEA). The rats were randomly assigned to the control (vehicle) or EGb (0.25, 0.50, and 1.00 g⋅kg^−1^) group (*n* = 6 per group).

### Drug

A standardized EGb, containing 24% flavonol glycosides, 5–7% terpene trilactones (2.8–3.4% ginkgolides A, B, C, J, M and 2.6–3.2% bilobalide), and <5 ppm ginkgolic acids provided by Galena Pharmaceutical, Campinas, Brazil) was used. EGb was re-suspended in water containing 12% Tween 80^®^. Single doses of EGb or vehicle solution were administered orally *via* a gastric tube 30 min prior to the acquisition session. The dose rational of choice in this work was based on previous work in our lab (Oliveira et al., 2009, 2013; Zamberlam et al., 2016) and published studies; several clinical studies have demonstrated that higher dosages (up to 600 mg day^−1^) appear to be necessary to enhance memory when an acute dosage is used (Allain et al., 1993; Le Bars and Kastelan, 2000). However, pre-clinical studies have demonstrated conflicting findings; however, a dose lower than 240–300 mg⋅kg^−1^ did not have an effect on cognitive performance when an acute dose (p.o) was administered. This finding is inconsistent with the findings reported for chronic treatment, in which a dose of 100 mg⋅kg^−1^ appears to be effective (Tchantchou et al., 2007; Blecharz-Klin et al., 2009; Yoshitake et al., 2010; Kehr et al., 2012).

### Behavioral Procedure

Conditioned suppression of licking responses was assessed as previously described (Oliveira et al., 2016; Zamberlam et al., 2016), and the assessments were conducted for 8 days. Briefly, prior to each experimental session, the rats were deprived of water for 12–16 h. On days 1–5, the rats were subjected to the acquisition of the lick response for 20 min during each session to obtain a stable baseline of drinking behavior. On day 6, each rat was returned to the experimental chamber, and the animals were subjected to four tone-shock (tone: 85 dB, 30 s; shock: 0.4 mA, 1.0 s) pairings; with the shock immediately following tone termination and a 5-min interval separating each successive pairing. On day 7, the animals were exposed to the experimental chamber, without stimuli presentation, to minimize the influence of context on this process and for the reacquisition of the licking response. On day 8, each rat was subjected to a retention test session, whereby the fear memory was retrieved *via* 10 successive conditioned stimulus (CS, tone) presentations (trials). The latencies to complete 10 licks prior to and during the tone were recorded and were used to calculate the suppression ratio (SR).

### Protein Sample Preparation

Twenty-four hours after the retention test ended, the animals were euthanized *via* decapitation, in the absence of anesthesia, and the dHF was rapidly removed, frozen in liquid nitrogen, and stored at −80°C until it was analyzed. Sample preparation was performed as previously described (Pedroso et al., 2012), with minor modifications. The entire dHF was homogenized in 1 mL of extraction buffer (7 M urea, 2 M thiourea, 4% CHAPS, 0.5% Triton X-100) containing cOmplete^TM^, Mini Protease Inhibitor Cocktail Tablets (Roche Diagnostics, Basel, Switzerland). Sample lysates were centrifuged at 4°C (14,000 rpm/30 min), and the supernatants were stored at −80°C until analysis. Protein concentrations of the supernatants were determined using a 2-D Quant Kit (GE Healthcare, Chicago, IL, United States) according to the manufacturer’s recommendations, using bovine albumin as a standard. The method applied to analysis of protein profile in this study was bottom-up proteomics file to aliquots of 750 μg of protein were precipitated with a solution of 35% chlorate potassium, 43% chloroform, and 22% methanol (v/v). The mixture was homogenized and centrifuged at 15,000 rpm and at 4°C for 15 min. The pellet was recovered and air-dried at room temperature.

### Two-Dimensional Gel Electrophoresis and Image Analysis

For isoelectric focusing (IEF), the pellet was dissolved in 500 μL of rehydration buffer [7 M urea, 2 M thiourea, 4% CHAPS, 0.5% Triton X-100, 100 mM DTT, 0.2% IPG buffer, pH 3–10 (GE Healthcare, United States), and traces of bromophenol blue]. IEF was carried out on a Protean^®^ IEF Cell (Bio-Rad, Hercules, CA, United States), using an Immobiline^TM^ DryStrip, with a pH of 3–10, an 18-cm linear gradient (GE Healthcare, United States), and having been previously rehydrated for 12–14 h. IEF was performed with the current limit set at 50 mA per IPG strip at 18°C with the following conditions: 250 V for 30 min, 500 V for 1 h, 1000 V for 1 h, 2000 V for 1 h, 4000 V for 1 h, and 8000 V for 1 h followed by 8000 V until 40,000 Vh. After focusing, the strips were equilibrated for 15 min in buffer containing 6 M urea, 2% SDS, 1.5 M Tris pH 8.8, 30% glycerol, and 1% DDT, followed by an additional 15 min in the same buffer containing 2.5% iodoacetamide instead of DTT. Strips were then loaded onto 12% SDS-polyacrylamide gels. After running in Protean^®^ II XL Multi-Cell (Bio-Rad, United States) at 60 mA per gel for 5 h, the gels were stained for 48 h with Coomassie Brilliant Blue G-250 (Bio-Rad, United States). The stained gels were scanned (GS-800^TM^ Calibrated Densitometer) and analyzed using the PDQuest^TM^ 2-D Analysis Software Version 8.0.1 (Bio-Rad, United States).

### Protein Identification

#### Protein Digestion

The protein spots that were quantitatively altered in response to treatment with EGb were manually excised from the gels, distained in 50% methanol and 2.5% acetic acid for 3 h, dehydrated with 100% acetonitrile, and dried in a vacuum concentrator. To the dried spots, 10 mM DTT was added, and the mixture was incubated for 30 min at room temperature, followed by a 50 mM iodoacetamide addition under the same conditions. The spots were washed and dehydrated with 100 mM ammonium bicarbonate and 100% acetonitrile, respectively, and were dried in vacuum concentrator. Digestion was performed overnight at 37°C with 20 ng/μL of trypsin (Promega, Fitchburg, WI, United States) in 50 mM ammonium bicarbonate. The digested samples were dried and re-suspended in 0.1% formic acid and were stored at −20°C until analysis (Shevchenko et al., 1996).

#### Mass Spectrometry Analysis

For the liquid chromatography–mass spectrometry analysis (LC–MS), peptides from the digested samples were injected onto a trap column (C18 trap column symmetry 180 μm × 20 mm, Waters, Milford, MA, United States) and were separated in the analytical column (C18 BEH 75 μm × 200 mm, 1.7 mm, Waters, United States) using a capillary UPLC system (nanoAcquity, Waters, United States). The elution gradient of 7–35% phase B (phase A: 0.1% formic acid in water, phase B: 0.1% formic acid in acetonitrile) was performed in 45 min at 275 nL/min. Multiple charged protonated peptides were generated by electrospray ionization and analyzed in a quadrupole time-of-flight mass spectrometer (Synapt HDMS G2, Waters, United States). Data were acquired in the MSE mode, switching from low (4 eV) to high (ramped from 19 to 45 eV) collision energy with scan times of 1.25 s. An external calibration was performed every 60 s using Glu-fibrinopeptide B (Waters, United States).

#### Data Analysis

Liquid chromatography–mass spectrometry analysis data were processed in the ProteinLynx Global SERVER^TM^ version 3.0.3 (Waters, United States) using low energy threshold of 750 counts and high-energy threshold of 50 counts. The MS/MS spectra were exported as mascot generic format (.mgf) files to MASCOT Server version 2.4 (Matrix Science, United Kingdom) for database search. Searches were performed in the UniprotKB ^1^ (92,378 sequences) and NCBI-nr ^2^ (92,378 sequences) protein databases. The following parameters were used in the searches: no restrictions on protein molecular weight; trypsin digest with up to one missing cleavage; monoisotopic mass; taxonomy limited to *Rattus*; carbamidomethylation of cysteine as a fixed modification; oxidation of methionine and tryptophan as variable modifications; a peptide mass tolerance of 10 ppm; and a fragment mass tolerance of 0.05 Da. The false discovery rate was estimated by the decoy database approach, and set to a maximum of 1%.

Protein matching probabilities were determined using the MASCOT protein scores, with the identification confidence indicated by the number of matches and by the coverage of the protein sequence by the matching peptides. The presence of at least one peptide with a significant ion score was required for positive protein identification. Only statistically significant MASCOT score results (*P* < 0.05) were included in the analysis.

### Statistical Analysis

The behavioral data were evaluated according to the mean SR of each trial for each rat daily. A two-way analysis of variance for repeated measures, followed by Tukey’s multiple comparisons test, was used to test for the presence of group and trial effects, as well as for the interaction between these variables. The optic densities of the spots were expressed as the percentage of change relative to the basal levels (vehicle) and were compared using one-way analysis of variance, followed by Tukey’s multiple comparisons test. The data were analyzed using GraphPad Prism version 7.00 (GraphPad Software, San Diego, CA, United States), and the significance level was 5%. All data are shown as the means ± standard error of the mean (see Supplementary Material).

### Interaction Network Analysis

Pathway analysis of the significantly altered optical density spots was performed using STRING^3^. Three protein–protein interaction networks (one for each EGb dose) of different spots were constructed with the confidence score set at 0.7 (high confidence).

## Results

### Behavioral Analysis

**FIGURE 1:**
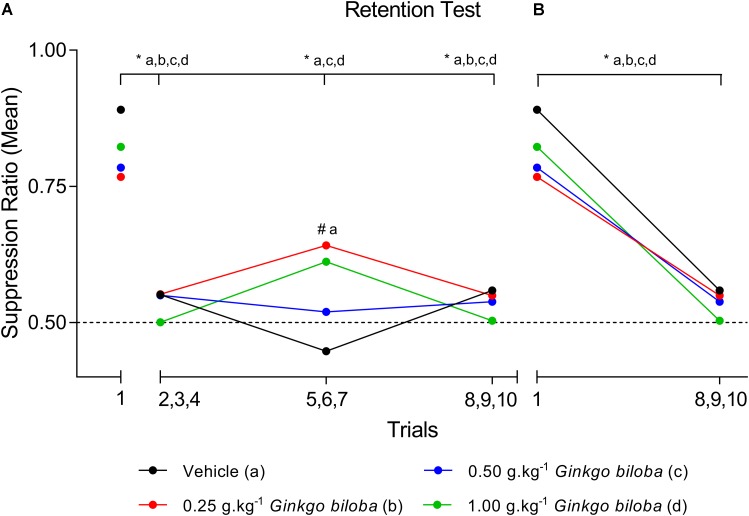
SR means of conditioned lick suppression during the retention test session (day 8). The points indicate the SR means of the first trial and the three-trial blocks for the control (vehicle) and EGb-treated (0.25, 0.50, and 1.00 g⋅kg^−1^) groups (*n* = 6 per group) **(A)** and first trial *versus* last three trial block **(B)**. Data represent mean. ^∗^*P* < 0.05 (two-way repeated measures ANOVA–Tukey *post hoc* test). ^#^Comparison among groups; ^∗^comparison intragroup.

Data regarding the first SR (SR_1_) means during the retention test demonstrated that all groups experienced the acquisition of conditioned suppression, namely, the EGb 0.25 g⋅kg^−1^ (SR_1_ = 0.77), 0.50 g⋅kg^−1^ (SR_1_ = 0.78), and 1.0 g⋅kg^−1^ (SR_1_ = 0.82) groups and the vehicle (SR_1_ = 0.89) group. Figure 1 shows SR means recorded across the ten trials of the memory retention sessions for the EGb-treated and vehicle groups, and all SRs are available in Supplementary Table S1. The first trial is presented independently because it represents the first presentation of the CS after conditioning and can characterize the level of fear of the animal in each situation. A two-way ANOVA analysis revealed no interaction between group × trial [*F*_(9,60)_ = 1.355; *P* = 0.2290] and a main effect of trial [*F*_(3,60)_ = 26.74; *P* < 0.0001] and no effect of group [*F*_(3,20)_ = 0.2604; *P* = 0.8531]. The comparisons between trials showed a significant decrease in the mean SR of the first trial compared with those of the three-trial blocks across all groups, except to group treated with 0.25 g⋅kg^−1^ dose during second three-trial block. Comparisons of the results for the first trial in the retention test sessions between groups revealed elevated SR in the subgroups treated with EGb and vehicle. The analysis of SR in the first three-trial block (second to fourth trials) showed a significant decrease in mean SR relative to the first trial in the vehicle and EGb groups. These results indicated the acquisition of extinction of fear memory within the session (Figure 1A). Furthermore, comparing the three-trial blocks revealed differences within sessions (*P* < 0.05). Rats treated with 0.25 g⋅kg^−1^ had return of fear during fifth to seventh trials of CS presentation. Furthermore, a reliable decrease in suppression and a reduction of fear to control and EGb at dose 0.5 g⋅kg^−1^. In summary, our data show that EGb did not prevent acquisition of lick suppression as well as within-session extinction (Figure 1B).

### Proteomics Analysis

The 2DE gels of the dHF (*n* = 5 per group) showed 344.0 ± 10.1 spots in the vehicle group and 355.6 ± 5.3 (0.25 g⋅kg^−1^), 361.6 ± 6.6 (0.50 g⋅kg^−1^), and 363.0 ± 4.7 (1.00 g⋅kg^−1^) spots in the EGb-treated groups. To analyze memory formation and the EGb treatment effect on protein expression, we analyzed the fold changes in protein expression between the control and EGb-treated groups. We identified 32 spots with significant changes in their optical density values (Table 1 and Supplementary Table S2). EGb treatment at all doses resulted in altered protein expression. Herein, six (0.25 g⋅kg^−1^), 12 (0.50 g⋅kg^−1^), and four (1.00 g⋅kg^−1^) proteins were upregulated, while six proteins and two proteins at each dose were downregulated in the EGb-treated groups in comparison to the vehicle group. The comparisons among the EGb-treated groups revealed that the intermediary dose (0.50 g⋅kg^−1^) positively affected protein expression in relation to the other doses. Ten proteins were found to be upregulated following treatment with 0.5 g⋅kg^−1^ EGb in relation to the lower dose (0.25 g⋅kg^−1^). Additionally, 13 proteins were upregulated, and two other proteins were downregulated in comparison to the group treated with 1.00 g⋅kg^−1^ EGb. Moreover, three proteins were significantly upregulated at the 1.00 g⋅kg^−1^ dosage, and three other proteins were downregulated in relation to the 0.25 g⋅kg^−1^ dose (Table 1).

**Table 1:** Proteins differentially expressed in the dorsal hippocampal formation of the rats treated with *Ginkgo biloba* (0.25, 0.50, and 1.0 g⋅kg^−1^) or vehicle groups and submitted to conditioned lick suppression.

Fold change (*P*-value)^1^						Protein description
0.25/vehicle^2^	0.50/vehicle^2^	1.00/vehicle^2^	0.50/0.25^2^	1.00/0.25^2^	1.00/0.50^2^	
3.27 (0.0247)						Dual specificity mitogen-activated protein kinase kinase 1
3.24 (0.0219)						Phosphoglycerate mutase 1
2.85 (0.0374)						Dihydropyrimidinase-related protein 5
4.30 (0.0120)						T-complex protein 1 subunit delta
	2.52 (0.0110)					Dihydropyrimidinase-related protein 2
	4.09 (0.0125)					Myosin-8
	1.95 (0.0098)	1.86 (0.0286)				Septin-6
			4.26 (0.0490)			Proteasome subunit alpha type-1
			4.09 (0.0126)			Vesicle-fusing ATPase
		6.11 (0.0020)		2.14 (0.0496)		Chain A, tetra-(5-fluorotryptophanyl)-glutathione transferase
		42.0 (0.0322)		12.4 (0.0336)	8.41 (0.0428)	Hypoxanthine-guanine phosphoribosyltransferase
					3.43 (0.0342)	40S ribosomal protein S14
0.56 (0.0255)	0.43 (0.0016)					Proteasome subunit alpha type-7
0.40 (0.0301)		0.47 (0.0458)				Endophilin-A1
	0.46 (0.0340)	0.51 (0.0464)				40S ribosomal protein SA
				0.35 (0.0333)		Synapsin-2
					0.32 (0.0488)	Beta-synuclein
					0.41 (0.0365)	Creatine kinase M-type
					0.46 (0.0378)	Transcriptional activator protein Pur-alpha
					0.46 (0.0032)	Myosin-6
2.61 (0.0225)				0.40 (0.0277)		WD repeat-containing protein 1
2.14 (0.0115)				0.35 (0.0039)		Dihydropyrimidinase-related protein 1
	2.16 (0.0411)				0.37 (0.0153)	MICOS complex subunit Mic60
	2.55 (0.0114)				0.41 (0.0213)	Myosin heavy chain IIa
	2.09 (0.0469)		2.72 (0.0210)		0.26 (0.0043)	14-3-3 protein eta
	2.38 (0.0250)		2.43 (0.0227)		0.36 (0.0130)	Heat shock protein HSP 90-alpha
	2.09 (0.0132)		1.89 (0.0262)		0.31 (0.0014)	Protein disulfide-isomerase A3
	2.06 (0.0082)		1.91 (0.0210)		0.58 (0.0301)	Hexokinase 1
	1.89 (0.0435)		2.69 (0.0098)		0.43 (0.0134)	Dynamin-1
	3.66 (0.0158)		2.50 (0.0362)		0.33 (0.0276)	Tubulin polymerization-promoting protein
			2.04 (0.0305)		0.47 (0.0235)	Myosin-4
	5.67 (0.0136)	6.77 (0.0044)	3.57 (0.0445)	4.27 (0.0142)		Proteasome subunit alpha type-6

*^1^A fold change value >1.0 indicates upregulation and a value of <1.0 indicates downregulation. P-value for Tukey post hoc test. ^2^The fold change was calculated as ratio of A/B where A is mean of group in the numerator and B mean of group in denominator.*

**FIGURE 2:**
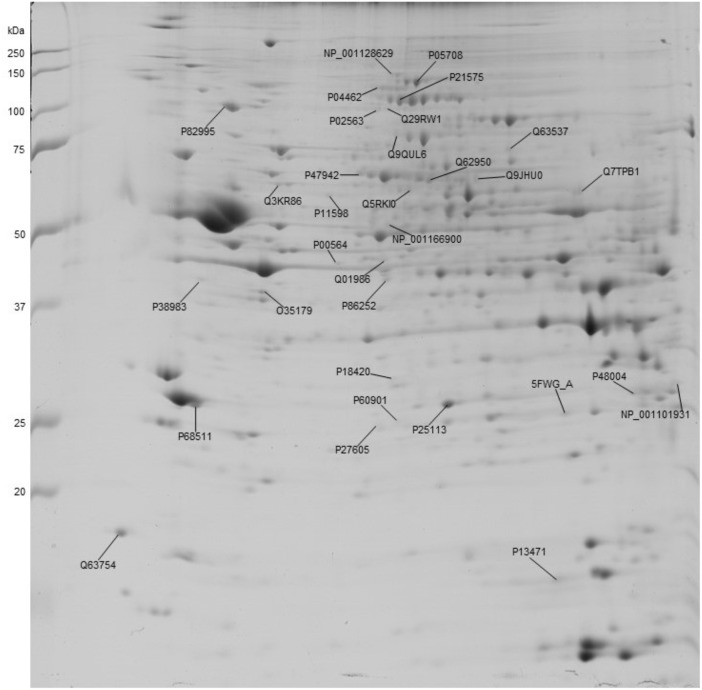
Representative image of a 2DE gel of a control (vehicle) dorsal hippocampal formation depicting the proteins that were significantly affected by EGb treatment. The numbers indicate the protein accession number.

Figure 2 shows a representative image of a 2DE gel of the vehicle group, with the indication of spots significantly affected by EGb treatment and submitted to conditioned lick suppression. The spots with significant optical density differences were analyzed using mass spectrometry for protein identification (Supplementary Table S3).

**FIGURE 3:**
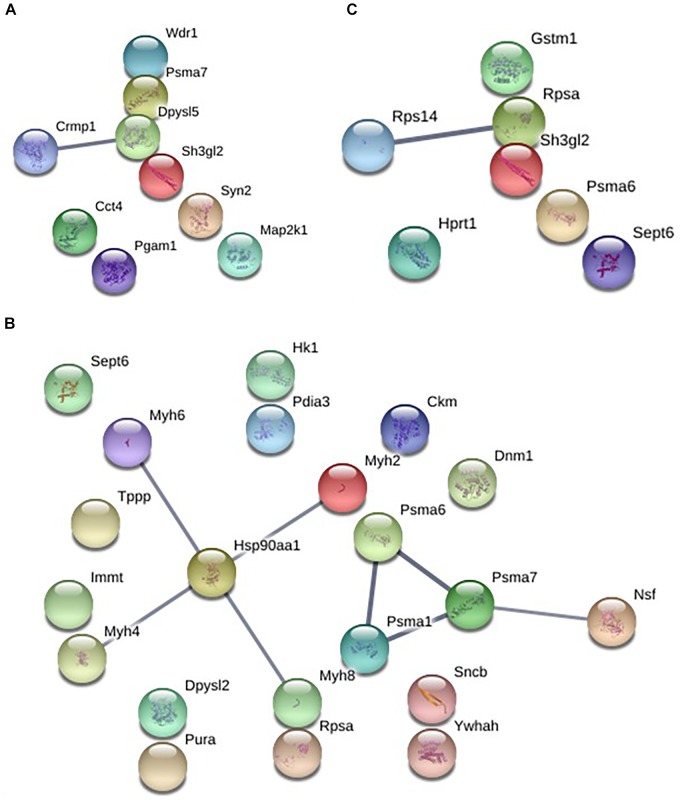
Graphic representation of the protein–protein interaction networks with significant differences between the groups in dorsal hippocampal formation. EGb 0.25 g⋅kg^−1^ (nine nodes), enrichment *P*-value = 0.127 **(A)**. EGb 0.50 g⋅kg^−1^ (21 proteins), enrichment *P*-value = 0.000335 **(B)**. EGb 1.00 g⋅kg^−1^ (seven proteins), enrichment *P*-value = 0.592 **(C)**.

The analysis of protein–protein interactions showed significant enrichments among the 21 proteins affected by EGb treatment at a dose of 0.50 g⋅kg^−1^ (*P* = 0.000335). On the other hand, treatment with EGb at doses of 0.25 g⋅kg^−1^ (nine nodes; *P* = 0.127) and 1.00 g⋅kg^−1^ (seven nodes; *P* = 0.592) indicated no significant interaction enrichments (Figure 3). The pathway analysis indicated that membrane-bound vesicles, cellular component morphogenesis, and cell projections were significantly modified following EGb treatment at a dose of 0.25 g⋅kg^−1^. Furthermore, treatment with EGb at a dose of 0.50 g⋅kg^−1^ modified pathways involved in processes related to heterocyclic compound binding, organic cyclic compound binding, and cell projection, as well as to the somatodendritic compartment, myelin sheath, neuronal cell body, tight junctions, and neurons. In addition, the group treated with EGb at the higher dose of 1.00 g⋅kg^−1^ exhibited modifications to the ribosomal small subunit assembly pathway (Table 2).

**Table 2:** Pathway significantly affected in the dorsal hippocampal formation of rats following treatment with standardized extract of *Ginkgo biloba* and acquisition of conditioned lick suppression.

	Pathway description	Protein	False discovery rate
0.25 g⋅kg^−1^ *Ginkgo biloba*	Membrane-bounded vesicle	T-complex protein 1 subunit delta ↑ Dual specificity mitogen-activated protein kinase kinase 1 ↑	0.0206
		Endophilin-A1 ↓	
		Synapsin-2 ↑	
		WD repeat-containing protein 1 ↑	
	Cellular component morphogenesis	Dihydropyrimidinase-related protein 1 ↑	0.0396
		Dihydropyrimidinase-related protein 5 ↑	
		Dual specificity mitogen-activated protein kinase kinase 1 ↑	
		WD repeat-containing protein 1 ↑	
	Cell projection	Dihydropyrimidinase-related protein 1 ↑	0.0429
		Dual specificity mitogen-activated protein kinase kinase 1 ↑	
		Synapsin-2 ↑	
		WD repeat-containing protein 1 ↑	
0.50 g⋅kg^−1^ *Ginkgo biloba*	Heterocyclic and organic cyclic compound binding	Creatine kinase M-type ↑ Dynamin-1 ↑	0.00615
		Hexokinase 1 ↑	
		Heat shock protein HSP 90-alpha ↑	
		Myosin-4 ↑	
		Myosin-6 ↑	
		Vesicle-fusing ATPase ↑	
		Protein disulfide-isomerase A3 ↑	
		Proteasome subunit alpha type-6 ↑	
	Cell projection part	Dynamin-1 ↑	0.00992
		Dihydropyrimidinase-related protein 2 ↑	
		Heat shock protein HSP 90-alpha ↑	
		Vesicle-fusing ATPase ↑	
		Beta-synuclein ↑	
	Somatodendritic compartment	Dihydropyrimidinase-related protein 2 ↑	0.0110
		Heat shock protein HSP 90-alpha ↑	
		Vesicle-fusing ATPase ↑	
		40S ribosomal protein SA ↓	
		Beta-synuclein ↑	
	Myelin sheath	Dihydropyrimidinase-related protein 2 ↑	0.0110
		Heat shock protein HSP 90-alpha ↑	
		Protein disulfide-isomerase A3 ↑	
	Neuronal cell body	Dihydropyrimidinase-related protein 2 ↑	0.0280
		Heat shock protein HSP 90-alpha ↑	
		40S ribosomal protein SA ↓	
		Beta-synuclein ↑	
	Tight junction	Myosin heavy chain IIa ↑	0.0305
		Myosin-4 ↑	
		Myosin-6 ↑	
	Neuron part	Dynamin-1 ↑	0.0499
		Heat shock protein HSP 90-alpha ↑	
		Vesicle-fusing ATPase ↑	
		40S ribosomal protein SA ↓	
		Beta-synuclein ↑	
1.00 g⋅kg^−1^ *Ginkgo biloba*	Ribosomal small subunit assembly	40S ribosomal protein S14 ↑ 40S ribosomal protein SA ↓	0.0300

*Up ↑ and down ↓ arrows indicate upregulation and downregulation, respectively. False discovery rate (corrected P-values for multiple testing using the method of Benjamini and Hochberg, 1995).*

## Discussion

The present data provided evidence of the modulatory effects of EGb on the acquisition of conditioned lick suppression by differential protein expression profiles in the dHF, corroborating our previous data (Oliveira et al., 2009; Zamberlam et al., 2016) and expanding our current knowledge about EGb effects on memory formation. Rats treated with EGb at all doses acquired suppression of the licking response and within-session extinction. However, our data revealed elevated SR in the subgroups treated with EGb at dose 0.25 g⋅kg^−1^ in relation to control group during second three-trial block (fifth to seventh), but in the subsequent trial, they had a reliable decrease in suppression and a reduction of fear, similar to all groups by the end of the session. Furthermore, comparisons between the first trial and the three-trial blocks showed reduced suppression of the licking response in the EGb at doses 0.5 and 1.0 g⋅kg^−1^, similar to the vehicle group. Our current findings corroborate with previous data from our lab about modulatory effects of EGb treatment on original memory, which was somewhat enhanced, i.e., better preserved, similar to found to the flavones from *Erythrina falcata* (Oliveira et al., 2016). These findings aligned with our hypothesis that EGb is able to modulate in a dose-dependent manner molecular mechanisms that underlie the acquisition/consolidation of fear memories and anxiety.

Acute doses of EGb have been demonstrated to enhance attention and memory in young healthy volunteers. A significant improvement in short-term memory has been demonstrated following *G. biloba* extract administration at a dose of 600 mg in volunteers with cognitive impairment (Allain et al., 1993; Le Bars and Kastelan, 2000) and healthy volunteers (Subhan and Hindmarch, 1984). Higher dosages (up to 600 mg⋅day^−1^) appear to be necessary to enhance memory when an acute dosage is used (Allain et al., 1993; Le Bars and Kastelan, 2000). One study demonstrated the efficacy of an acute treatment of EGb with 240 and 360 mg in young volunteers (Kennedy et al., 2000). Furthermore, EGb at a daily dose of 240 mg (chronic treatment) has been considered necessary to successfully ameliorate clinical symptoms, such as apathy, depression, motor alterations, and cognitive deficits (Gavrilova et al., 2014). Additionally, effects of EGb on mitochondrial function might substantiate their function on cognition and ameliorate the age-associated cognitive disorders (Müller et al., 2017).

In rats, several studies have showed that a lower than 240–300 mg⋅kg^−1^ did not have effect on cognitive performance when acute dose is administered, different than observed to chronic treatment (Tchantchou et al., 2007; Blecharz-Klin et al., 2009; Yoshitake et al., 2010; Kehr et al., 2012). Subacute treatment with EGb, at dose 300 mg⋅kg^−1^ was a significant increase in extracellular monoamines in pre-frontal cortex was related (5HT, Ach, and DOPA), which was considered relevant to clinical doses (Yoshitake et al., 2010; Kehr et al., 2012). Nevertheless, studies that evaluated the effects of chronic treatment of EGb in mice AD model showed that plasma concentration of EGb metabolites after chronic administration (5 months) of diet supplementary with EGb in dose around 69 mg⋅kg^−1^⋅day^−1^), was similar to secondary metabolites found in plasma human after with chronic treatment with 240 mg⋅day^−1^ EGb. Further chronic treatment with EGb 761 at dose 240–480 mg was effective in reduces anxiety in middle-age patients with anxiety disorders, but in higher dose, the effects were more pronounced (Woelk et al., 2007). In this study, a dose of 500 mg⋅kg^−1^ was effective in reduce a conditioned suppression (anxiety-like effects) without impair fear memory. A comparative analysis between specific effects of EGb *in vivo* from pre clinical studies and equivalent dosage for humans was showed for Serrano-García et al. (2013), but all studies evaluate was for subacute or chronic treatment. In recent study, Tan et al. (2015) showed that treatment chronic with EGb was a be able to stabilize or slow decline in cognition function, global function, and behavior symptoms in patients with neuropsychiatric symptoms. Conversely, Birks and Colleagues (Birks and Evans, 2009; Yuan et al., 2017) showed in a meta-analysis study, that treatments of EGb seems no correlated with dose only. Furthermore, in attempt to evaluate drugs safety and efficacy, our group evaluate the suppression of licking behavior in rats submitted to acute, subacute, and chronic treatment with EGb (Oliveira et al., 2009, 2013), that results was compared with another anti-anxiety drugs. Acute (one dose), subacute (14 days), or chronic (30 days) treatment with EGb at dose of 500 and 1000 mg⋅kg^−1^ did not impair acquisition of fear memory. Furthermore, although the dose and regime of treatments were different, we found similar effects on gene expression in prefrontal cortex, amygdala, and hippocampus. However, in higher dose the effects were more pronounced.

We depicted for the first time that EGb modulates 32 proteins through both upregulation and downregulation in the dHF in a dose-dependent manner. We also found that treatment with EGb at a dose of 0.50 g⋅kg^−1^ affected the expression of a greater number of regulatory proteins when compared with the other doses. Furthermore, our results suggested that EGb modulates proteins, which might have therapeutic relevance since that they may be modulated for new pharmacological agents that can be used to enhance memory formation and to prevent or treat memory decline or anxiety disorders. To understand the differential effects of EGb, we investigated the protein–protein interactions at each dose.

Memory formation and its maintenance might involve distinct processes that occur over time and both *de novo* protein synthesis and post-translational modification of existing proteins (Jarome and Helmstetter, 2014; Yau et al., 2015). In this context, our present findings corroborated those of a large number of studies showing that molecular changes underlie memory acquisition and consolidation and have since shown that acquisition of conditioned lick suppression and treatment with EGb are correlated with multiple biological processes and signaling pathways that are crucial to the formation and maintenance of LTM. The differential protein expression patterns recognized in our studies have been found to be correlated with dendritic spine expansion or projection, the composition of structures in the somatodendritic compartment of neurons, the proteins involved in myelin sheath formation, and tight-junction composition.

The upregulated expression of dynamin-1, vesicle-fusing ATPase, and dihydropyrimidinase-related protein 2 in the dHF after treatment with EGb at a dose of 0.5 g⋅kg^−1^ suggested that EGb modulates events correlated to remodeling of the cytoskeleton and those involved in the control of the distinct pathways involved in neurotransmission events since they are also involved in dendritic spine development and maintenance, synaptic vesicle recycling, and insertion of glutamatergic receptors at synaptic sites, which are events that are described as essential to LTM formation and neurogenesis. Still, changes in these protein expression levels have been found to be correlated with hippocampal-dependent memory formation (Beretta et al., 2005; Guo et al., 2010; Fà et al., 2014; Migues et al., 2014; Jin et al., 2016; Yoo et al., 2016; Zhang et al., 2016). Corroborating with previous study, our data showed that EGb treatment modulate proteins whose activity are essential for normal mitochondrial function and synaptic plasticity in hippocampus as Dynanin-1 (Shields et al., 2015) and proteins, as Hexokinase 1 (HKI), which is associated with the outer mitochondrial membrane. HKI activity in neurons has been associated with cell survival and neuroprotection by suppressing apoptosis and oxidative stress (Saraiva et al., 2010).

Our results showed that the 0.50 g⋅kg^−1^ dose of EGb upregulated heat shock protein 90 alpha, disulfide-isomerase A3, and beta-synuclein, which are important in cellular homeostasis since they are involved in the transient changes in the activity of specific proteins that participate in the intracellular cascades that regulate gene transcription and *de novo* protein synthesis in the brain, cell stress responses, synaptic transmission, and autophagy (Stetler et al., 2010; Jarome and Helmstetter, 2014; Yau et al., 2015).

Several studies have noted that the heat shock protein 90 family facilitates cell signaling; assists in the efficient folding of newly translated proteins intracellular transport, maintenance and degradation of proteins; and the protection of mesenchymal stem cells from apoptosis and stimulates their migration. Furthermore, their roles in calcium homeostasis, neuron survival, axonal regeneration, and neuroprotection of the central and peripheral nervous systems have been shown (Loones et al., 2000; Gao et al., 2015; Ousman et al., 2017). The pharmacological inhibition of HSP 90 has been associated with neurodegenerative disease, suggesting the protective role of these proteins (Luo et al., 2010). Recent evidence has suggested their role in the enhancement of memory formation (Gyurko et al., 2014). Another chaperone protein that is important in the quality control of protein folding is disulfide-isomerase A3, which is an enzyme in the endoplasmic reticulum of eukaryotic cells that acts as a binding partner for other proteins and has a role in myelin sheath preservation. Their therapeutic roles as neuroprotective/anti-apoptotic and prosurvival proteins in several neurological disorders, including Alzheimer’s disease, have been investigated (Hoffstrom et al., 2010; Imaoka, 2011; Gonzalez-Perez et al., 2015). Regarding beta-synuclein, its role in the central nervous system remains unclear. Beta-synuclein has been found to be associated with membrane stability and/or turnover of membrane components, and it also might act as a chaperone and might be found in pre-synaptic nerve terminals that are presently thought to be important for neural plasticity (George, 2001; Mori et al., 2002; Fujita et al., 2006). Their role in neurodegenerative diseases, such as Alzheimer’s, Parkinson’s, or ischemic disease, has been evaluated (Tanaka et al., 2000; Hashimoto et al., 2004; Hoffstrom et al., 2010; Leak, 2014).

Therefore, the upregulation of these proteins following treatment with EGb at a dose of 0.50 g⋅kg^−1^ aligned with the antioxidant and neuroprotective effects that have been proposed for EGb (DeFeudis, 2002; Ahlemeyer and Krieglstein, 2003a; Ribeiro et al., 2016) and indicated a possible therapeutic use of this extract in the prevention of neural diseases. Furthermore, previous data from our group showed that conditioned lick suppression downregulated alpha-synuclein (Gaiardo et al., unpublished) and that EGb upregulated beta-synuclein, which has been recognized as playing a role in chaperone activity more efficiently than alpha-synuclein (Lee et al., 2004). The correlation between the presence of beta-synuclein and the significant reduction in the rate of alpha-synuclein aggregation was shown by Brown et al. (2016), suggests a neuroprotective effect.

Myosin IIA is less predominant in the central nervous system, is required in the maintenance of tensile adhesion and neurite retraction, and is regulated during the induction of long-term potentiation. Their actions combine to generate the vectorial forces that are required for neurite extension (Wylie and Chantler, 2003; Liu and Cheney, 2012; Luissint et al., 2012). Myosin IIA was found to have a stronger association with actin filaments under oxidative stress, and the inhibition of the myosin IIA–actin interaction was found to be correlated with attenuated apoptosis and enhanced survival of PC12 neural cells in culture (Wang et al., 2017) and modulates synaptic plasticity in the lateral amygdaloid complex, which is involved in fear memory formation, seemingly preventing irrelevant memories (Lamprecht et al., 2006). Myosin VI is found in Golgi complexes and has a higher affinity for ADP. It is involved in the endocytosis and phagocytosis of AMPA receptors and serves as a target for other proteins involved in neurodegenerative disorders, such as Huntington’s disease (Osterweil et al., 2005). Similarly, we observed upregulation of myosin IV following treatment with EGb at a dose of 0.5 g⋅kg^−1^. Both myosin IIA and VI play roles in calcium binding and protein phosphorylation (Buss and Kendrick-Jones, 2008).

Regarding the role of myosin, the present data allowed us to suggest that EGb might act by enabling the neural network to become more stable since it was found to promote changes in the cellular morphology and physiology of neural cells *via* changes in the activity of the different isoforms of myosin.

Concerning the effects on rats treated with EGb at a dose 0.25 g⋅kg^−1^, we found that EGb modulates different pathways and proteins, and it may have the same effects *via* other pathways, for example, upregulation of proteins such as dual specificity mitogen-activated protein kinase 1, synapsin-2, dihydropyrimidinase-related protein 5, dihydropyrimidinase-related protein 1, and T-complex protein 1 subunit delta (molecular chaperone). These proteins have a crucial role in the control of signaling in long-lasting forms of synaptic plasticity and memory, modulating neurotransmitter release at the pre-synaptic cell, and playing a role in the generation and survival of newly generated neurons in the areas of the adult brain with a high level of activity-dependent neuronal plasticity (Kelleher et al., 2004; Bretin et al., 2005; Cesca et al., 2010).

The analysis of protein expression in the dHFs of rats treated with EGb at a dose of 1.0 g⋅kg^−1^ revealed an upregulation of septin-6, a member of a protein family that is highly expressed in the brain and takes part in processes such as regulation of the formation, growth and stability of axons and dendrites, synaptic plasticity, and vesicular trafficking, which are essential for memory formation (Cho et al., 2011; Hall and Russell, 2012). In addition, EGb treatment in higher doses may promote protective and preventive effects since proteasome 6 and tetra-(5-fluorotryptophanyl)-glutathione-S-transferase (all four tryptophan residues are replaced by the synthetic amino acid 5-fluorotryptophan, which causes a modest increase in catalytic activity) were upregulated. Further analyses are necessary, but these proteins might result in the improvement of fear memory consolidation and in the spontaneous recovery of fear memory established at this dose in previous studies by our group (Zamberlam et al., 2016).

Protein degradation is a stage in protein turnover regulation. Studies over the last decade have demonstrates strong links between the maintenance of long-term potentiation and protein degradation (Dong et al., 2008; Hegde, 2010). Several behavioral studies have also confirmed the crucial role of the ubiquitin-proteasome system (UPS) in memory consolidation in the hippocampal formation (Lopez-Salon et al., 2001; Artinian et al., 2008). The effects of dysregulation of the UPS on neurons and glial cells may contribute to several neural diseases because large insoluble aggregates of misfolded proteins can form and then result in neurotoxicity (Lehman, 2009; Jansen et al., 2014). These proteins are essential for the disposal of exogenous toxic compounds and for antioxidant responses to reactive oxygen species (Strange et al., 2001). Our findings showed upregulation of the alpha type subunits (1 and 6) of the UPS, corroborating the findings of previous studies, which showed upregulation of the UPS following EGb treatment (Liu et al., 2009; Stark and Behl, 2014).

Furthermore, proteasome subunit alpha type-7, endophilin-A1, and 40S ribosomal protein SA were downregulated following EGb treatment in comparison with the vehicle group. Despite the reduction observed in these proteins, other proteins with the same effects were upregulated. For example, proteasome subunit alpha type-7 was downregulated by the 0.25 and 0.50 g⋅kg^−1^ doses; conversely, these doses upregulated other proteins with similar functions, such as T-complex protein 1 subunit delta, proteasome subunit alpha type-1, and heat shock protein HSP 90-alpha. In this context, EGb modulates, in a dose-dependent manner, different pathways, which may correlate with the behavioral effects that were previously described by our group in relation to fear memory and anxiety.

In summary, the comparative dHF proteome analysis allowed us to identify proteins whose altered expression may underlie the effects of EGb on conditioned suppression. We identified 32 different protein expression patterns that correlated with dendritic spine expansion or projection, the composition of structures in the somatodendritic compartment, and with proteins involved in myelin sheath formation and preservation, as well as those involved in tight-junction composition, which are mechanisms that are involved in the long-term changes that are crucial for LTM formation. The present data might explain, at least in part, the beneficial effects of EGb on memory formation.

## Proteome Profiling Data

The mass spectrometry proteomics data have been deposited to the PRIDE Archive (http://www.ebi.ac.uk/pride/archive/) *via* the PRIDE partner repository with the data set identifier PXD009894.

## Author Contributions

RG performed all the experiments and was responsible for the writing of the manuscript in its entirety. TA performed the mass spectrometry analysis. SC was responsible for conceptualizing and revising the manuscript. AT and MT were involved in conceptualizing and proofreading. All authors gave their final approval for the submission of the manuscript.

## Conflict of Interest Statement

The authors declare that the research was conducted in the absence of any commercial or financial relationships that could be construed as a potential conflict of interest.

## Footnotes

1 www.uniprot.org

2 www.ncbi.nlm.nih.gov/protein

3 http://string-db.org/

## References

[B1] Ahlemeyer B. Krieglstein J. (2003a). Neuroprotective effects of *Ginkgo biloba* extract. *Cell Mol. Life Sci. C* 60 1779–1792. 10.1007/s00018-003-3080-1 14523543 PMC11146048

[B2] Ahlemeyer B. Krieglstein J. (2003b). Pharmacological studies supporting the therapeutic use of *Ginkgo biloba* extract for Alzheimer’s disease. *Pharmacopsychiatry* 36 8–14. 10.1055/s-2003-40454 13130383

[B3] Allain H. Raoul P. Lieury A. LeCoz F. Gandon J. M. d’Arbigny P. (1993). Effect of two doses of *Ginkgo biloba* extract (EGb 761) on the dual-coding test in elderly subjects. *Clin. Ther.* 15 549–558. 8364946

[B4] Artinian J. McGauran A.-M. T. De Jaeger X. Mouledous L. Frances B. Roullet P. (2008). Protein degradation, as with protein synthesis, is required during not only long-term spatial memory consolidation but also reconsolidation. *Eur. J. Neurosci.* 27 3009–3019. 10.1111/j.1460-9568.2008.06262.x 18588539

[B5] Barco A. Pittenger C. Kandel E. R. (2003). CREB, memory enhancement and the treatment of memory disorders: promises, pitfalls and prospects. *Expert Opin. Ther. Targets* 7 101–114. 10.1517/14728222.7.1.101 12556206

[B6] Benjamini Y. Hochberg Y. (1995). Controlling the false discovery rate: a practical and powerful approach to multiple testing. *J. R. Stat. Soc.* 57 289–300. 10.1111/j.2517-6161.1995.tb02031.x

[B7] Beretta F. Sala C. Saglietti L. Hirling H. Sheng M. Passafaro M. (2005). NSF interaction is important for direct insertion of GluR2 at synaptic sites. *Mol. Cell. Neurosci.* 28 650–660. 10.1016/j.mcn.2004.11.008 15797712

[B8] Birks J. Evans J. G. (2009). *Ginkgo biloba* for cognitive impairment and dementia. *Cochr. Database Syst. Rev.* 18:CD003120. 10.1002/14651858.CD003120.pub3 19160216 PMC13076002

[B9] Blecharz-Klin K. Piechal A. Joniec I. Pyrzanowska J. Widy-Tyszkiewicz E. (2009). Pharmacological and biochemical effects of *Ginkgo biloba* extract on learning, memory consolidation and motor activity in old rats. *Acta Neurobiol. Exp. (Wars)* 69 217–231. 19593336 10.55782/ane-2009-1747

[B10] Bretin S. Reibel S. Charrier E. Maus-Moatti M. Auvergnon N. Thevenoux A. (2005). Differential expression of CRMP1, CRMP2A, CRMP2B, and CRMP5 in axons or dendrites of distinct neurons in the mouse brain. *J. Comp. Neurol.* 486 1–17. 10.1002/cne.20465 15834957

[B11] Brown J. W. P. Buell A. K. Michaels T. C. T. Meisl G. Carozza J. Flagmeier P. (2016). β-Synuclein suppresses both the initiation and amplification steps of α-synuclein aggregation via competitive binding to surfaces. *Sci. Rep.* 6:36010. 10.1038/srep36010 27808107 PMC5093550

[B12] Buss F. Kendrick-Jones J. (2008). How are the cellular functions of myosin VI regulated within the cell? *Biochem. Biophys. Res. Commun.* 369 165–175. 10.1016/j.bbrc.2007.11.150 18068125 PMC2635068

[B13] Cammarota M. Bevilaqua L. R. Rossato J. I. Lima R. H. Medina J. H. Izquierdo I. (2008). Parallel memory processing by the CA1 region of the dorsal hippocampus and the basolateral amygdala. *Proc. Natl. Acad. Sci. U.S.A.* 105 10279–10284. 10.1073/pnas.0805284105 18647831 PMC2492498

[B14] Carew T. J. Sahley C. L. (1986). Invertebrate learning and memory: from behavior to molecules. *Annu. Rev. Neurosci.* 9 435–487. 10.1146/annurev.ne.09.030186.002251 2423010

[B15] Cerutti S. M. Gomide V. C. de Moraes Ferrari E. A. Chadi G. (2009). Long-term astroglial reaction and neuronal plasticity in the subcortical visual pathways after a complete ablation of telencephalon in pigeons (*Columba livia*). *Int. J. Neurosci.* 119 384–403. 10.1080/00207450802480291 19116845

[B16] Cesca F. Baldelli P. Valtorta F. Benfenati F. (2010). The synapsins: key actors of synapse function and plasticity. *Prog. Neurobiol.* 91 313–348. 10.1016/j.pneurobio.2010.04.006 20438797

[B17] Cho S.-J. Lee H. Dutta S. Song J. Walikonis R. Moon I. S. (2011). Septin 6 regulates the cytoarchitecture of neurons through localization at dendritic branch points and bases of protrusions. *Mol. Cells* 32 89–98. 10.1007/s10059-011-1048-9 21544625 PMC3887662

[B18] DeFeudis F. V. (2002). Effects of *Ginkgo biloba* extract (EGb 761) on gene expression: possible relevance to neurological disorders and age-associated cognitive impairment. *Drug Dev. Res.* 57 214–235. 10.1002/ddr.10151

[B19] Dong C. Upadhya S. C. Ding L. Smith T. K. Hegde A. N. (2008). Proteasome inhibition enhances the induction and impairs the maintenance of late-phase long-term potentiation. *Learn. Mem.* 15 335–347. 10.1101/lm.984508 18441292 PMC2364605

[B20] Fà M. Staniszewski A. Saeed F. Francis Y. I. Arancio O. (2014). Dynamin 1 is required for memory formation. *PLoS One* 9:e91954. 10.1371/journal.pone.0091954 24643165 PMC3958425

[B21] Fendt M. Fanselow M. S. (1999). The neuroanatomical and neurochemical basis of conditioned fear. *Neurosci. Biobehav. Rev.* 23 743–760. 10.1016/S0149-7634(99)00016-0 10392663

[B22] Frey U. Huang Y. Kandel E. (1993). Effects of cAMP simulate a late stage of LTP in hippocampal CA1 neurons. *Science (80-)* 260 1661–1664. 10.1126/science.8389057 8389057

[B23] Fujita M. Wei J. Nakai M. Masliah E. Hashimoto M. (2006). Chaperone and anti-chaperone: two-faced synuclein as stimulator of synaptic evolution. *Neuropathology* 26 383–392. 10.1111/j.1440-1789.2006.00732.x 17080714

[B24] Gao F. Hu X. Xie X. Liu X. Wang J. (2015). Heat shock protein 90 stimulates rat mesenchymal stem cell migration via PI3K/Akt and ERK1/2 pathways. *Cell Biochem. Biophys.* 71 481–489. 10.1007/s12013-014-0228-6 25287672

[B25] Gavrilova S. I. Preuss U. W. Wong J. W. M. Hoerr R. Kaschel R. Bachinskaya N. (2014). Efficacy and safety of *Ginkgo biloba* extract EGb 761? in mild cognitive impairment with neuropsychiatric symptoms: a randomized, placebo-controlled, double-blind, multicenter trial. *Int. J. Geriatr. Psychiatry* 29 1087–1095. 10.1002/gps.4103 24633934

[B26] George J. M. (2001). The synucleins. *Genome Biol.* 3:reviews3002.1. 10.1186/gb-2001-3-1-reviews3002 PMC150459 11806835

[B27] Giese K. P. Mizuno K. (2013). The roles of protein kinases in learning and memory. *Learn. Mem.* 20 540–552. 10.1101/lm.028449.112 24042850

[B28] Gonzalez-Perez P. Woehlbier U. Chian R.-J. Sapp P. Rouleau G. A. Leblond C. S. (2015). Identification of rare protein disulfide isomerase gene variants in amyotrophic lateral sclerosis patients. *Gene* 566 158–165. 10.1016/j.gene.2015.04.035 25913742 PMC5553116

[B29] Guo C.-H. Senzel A. Li K. Feng Z.-P. (2010). De novo protein synthesis of syntaxin-1 and dynamin-1 in long-term memory formation requires CREB1 gene transcription in *Lymnaea stagnalis*. *Behav. Genet.* 40 680–693. 10.1007/s10519-010-9374-9 20563839

[B30] Gyurko D. M. Soti C. Stetak A. Csermely P. (2014). System level mechanisms of adaptation, learning, memory formation and evolvability: the role of chaperone and other networks. *Curr. Protein Pept. Sci.* 15 171–188. 10.2174/1389203715666140331110522 24694371

[B31] Hall P. A. Russell S. H. (2012). Mammalian septins: dynamic heteromers with roles in cellular morphogenesis and compartmentalization. *J. Pathol.* 226 287–299. 10.1002/path.3024 21990096

[B32] Hashimoto M. Bar-on P. Ho G. Takenouchi T. Rockenstein E. Crews L. (2004). β-synuclein regulates Akt activity in neuronal cells. *J. Biol. Chem.* 279 23622–23629. 10.1074/jbc.M313784200 15026413

[B33] Hegde A. N. (2010). The ubiquitin-proteasome pathway and synaptic plasticity. *Learn. Mem.* 17 314–327. 10.1101/lm.1504010 20566674 PMC2904103

[B34] Hoffstrom B. G. Kaplan A. Letso R. Schmid R. S. Turmel G. J. Lo D. C. (2010). Inhibitors of protein disulfide isomerase suppress apoptosis induced by misfolded proteins. *Nat. Chem. Biol.* 6 900–906. 10.1038/nchembio.467 21079601 PMC3018711

[B35] Hotulainen P. Hoogenraad C. C. (2010). Actin in dendritic spines: connecting dynamics to function. *J. Cell Biol.* 189 619–629. 10.1083/jcb.201003008 20457765 PMC2872912

[B36] Imaoka S. (2011). “Chemical stress on protein disulfide isomerases and inhibition of their functions,” in *International Review of Cell and Molecular Biology*, ed. Jeon K. W. (Amsterdam: Elsevier Inc.), 121–166. 10.1016/B978-0-12-386037-8.00003-X 21875564

[B37] Itil T. M. Eralp E. Tsambis E. Itil K. Z. Stein U. (1996). Central nervous system effects of *Ginkgo biloba*, a plant extract. *Am. J. Ther.* 3 63–73. 10.1097/00045391-199601000-00009 11856998

[B38] Izquierdo I. Quillfeldt J. A. Zanatta M. S. Quevedo J. Schaeffer E. Schmitz P. K. (1997). Sequential role of hippocampus and amygdala, entorhinal cortex and parietal cortex in formation and retrieval of memory for inhibitory avoidance in rats. *Eur. J. Neurosci.* 9 786–793. 10.1111/j.1460-9568.1997.tb01427.x 9153585

[B39] Jansen A. H. P. Reits E. A. J. Hol E. M. (2014). The ubiquitin proteasome system in glia and its role in neurodegenerative diseases. *Front. Mol. Neurosci.* 7:73 10.3389/fnmol.2014.00073 PMC4126450 25152710

[B40] Jarome T. J. Helmstetter F. J. (2014). Protein degradation and protein synthesis in long-term memory formation. *Front. Mol. Neurosci.* 7:61 10.3389/fnmol.2014.00061 PMC4072070 25018696

[B41] Jin X. Sasamoto K. Nagai J. Yamazaki Y. Saito K. Goshima Y. (2016). Phosphorylation of CRMP2 by Cdk5 regulates dendritic spine development of cortical neuron in the mouse hippocampus. *Neural Plast.* 2016 1–7. 10.1155/2016/6790743 26819770 PMC4706976

[B42] Kandel E. R. (2001). The molecular biology of memory storage: a dialogue between genes and synapses. *Science (80-)* 294 1030–1038. 10.1126/science.1067020 11691980

[B43] Kandel E. R. Dudai Y. Mayford M. R. (2014). The molecular and systems biology of memory. *Cell* 157 163–186. 10.1016/j.cell.2014.03.001 24679534

[B44] Kehr J. Yoshitake S. Ijiri S. Koch E. Nöldner M. Yoshitake T. (2012). *Ginkgo biloba* leaf extract (EGb 761^®^) and its specific acylated flavonol constituents increase dopamine and acetylcholine levels in the rat medial prefrontal cortex: possible implications for the cognitive enhancing properties of EGb 761^®^. *Int. Psychogeriatr.* 24(Suppl. 1), S25–S34. 10.1017/S1041610212000567 22784425

[B45] Kelleher R. J. Govindarajan A. Jung H.-Y. Kang H. Tonegawa S. (2004). Translational control by MAPK signaling in long-term synaptic plasticity and memory. *Cell* 116 467–479. 10.1016/S0092-8674(04)00115-1 15016380

[B46] Kennedy D. O. Scholey A. B. Wesnes K. A. (2000). The dose-dependent cognitive effects of acute administration of *Ginkgo biloba* to healthy young volunteers. *Psychopharmacology (Berl)* 151 416–423. 10.1007/s002130000501 11026748

[B47] Lamprecht R. Margulies D. S. Farb C. R. Hou M. Johnson L. R. LeDoux J. E. (2006). Myosin light chain kinase regulates synaptic plasticity and fear learning in the lateral amygdala. *Neuroscience* 139 821–829. 10.1016/j.neuroscience.2005.12.055 16515842

[B48] Le Bars P. L. Kastelan J. (2000). Efficacy and safety of a *Ginkgo biloba* extract. *Public Heal Nutr.* 3 495–499. 10.1017/S1368980000000574 11276297

[B49] Leak R. K. (2014). Heat shock proteins in neurodegenerative disorders and aging. *J. Cell Commun. Signal.* 8 293–310. 10.1007/s12079-014-0243-9 25208934 PMC4390795

[B50] Lee D. Paik S. R. Choi K. Y. (2004). β-Synuclein exhibits chaperone activity more efficiently than α-synuclein. *FEBS Lett.* 576 256–260. 10.1016/j.febslet.2004.08.075 15474047

[B51] Lehman N. L. (2009). The ubiquitin proteasome system in neuropathology. *Acta Neuropathol.* 118 329–347. 10.1007/s00401-009-0560-x 19597829 PMC2716447

[B52] Liu K. C. Cheney R. E. (2012). Myosins in cell junctions. *Bioarchitecture* 2 158–170. 10.4161/bioa.21791 22954512 PMC3696060

[B53] Liu X.-P. Goldring C. E. P. Wang H.-Y. Copple I. M. Kitteringham N. R. Park B. K. (2009). Extract of *Ginkgo biloba* induces glutathione-S-transferase subunit-P1 in vitro. *Phytomedicine* 16 451–455. 10.1016/j.phymed.2008.11.001 19131229

[B54] Loones M.-T. Chang Y. Morange M. (2000). The distribution of heat shock proteins in the nervous system of the unstressed mouse embryo suggests a role in neuronal and non-neuronal differentiation. *Cell Stress Chaper.* 5:291. 10.1379/1466-1268(2000)005<0291:TDOHSP>2.0.CO;2 11048652 PMC312859

[B55] Lopez-Salon M. Alonso M. Vianna M. R. M. Viola H. E. Souza T. M. Izquierdo I. (2001). The ubiquitin-proteasome cascade is required for mammalian long-term memory formation. *Eur. J. Neurosci.* 14 1820–1826. 10.1046/j.0953-816x.2001.01806.x 11860477

[B56] Luissint A.-C. Artus C. Glacial F. Ganeshamoorthy K. Couraud P.-O. (2012). Tight junctions at the blood brain barrier: physiological architecture and disease-associated dysregulation. *Fluids Barriers CNS* 9:23. 10.1186/2045-8118-9-23 23140302 PMC3542074

[B57] Luo W. Sun W. Taldone T. Rodina A. Chiosis G. (2010). Heat shock protein 90 in neurodegenerative diseases. *Mol. Neurodegener.* 5:24. 10.1186/1750-1326-5-24 20525284 PMC2896944

[B58] Lynch G. (2002). Memory enhancement: the search for mechanism-based drugs. *Nat. Neurosci.* 5(Suppl.), 1035–1038. 10.1038/nn935 12403980

[B59] Mayford M. Siegelbaum S. A. Kandel E. R. (2012). Synapses and memory storage. *Cold Spring Harb. Perspect. Biol.* 4 1–18. 10.1101/cshperspect.a005751 22496389 PMC3367555

[B60] McGaugh J. L. (1966). Time-dependent processes in memory storage. *Science (80-)* 153 1351–1358. 10.1126/science.153.3742.1351 5917768

[B61] Migues P. V. Hardt O. Finnie P. Wang Y. W. Nader K. (2014). The maintenance of long-term memory in the hippocampus depends on the interaction between N -ethylmaleimide-sensitive factor and GluA2. *Hippocampus* 24 1112–1119. 10.1002/hipo.22295 24753224

[B62] Mori F. Tanji K. Yoshimoto M. Takahashi H. Wakabayashi K. (2002). Immunohistochemical comparison of α- and β-synuclein in adult rat central nervous system. *Brain Res.* 941 118–126. 10.1016/S0006-8993(02)02643-4 12031554

[B63] Müller W. E. Eckert A. Eckert G. P. Fink H. Friedland K. Gauthier S. (2017). Therapeutic efficacy of the Ginkgo special extract EGb761^®^ within the framework of the mitochondrial cascade hypothesis of Alzheimer’s disease. *World J. Biol. Psychiatry* [Epub ahead of print]. 10.1080/15622975.2017.1308552 28460580

[B64] Oliveira D. R. de Zamberlam C. R. Rêgo G. M. Cavalheiro A. J. Cerutti J. M. Cerutti S. M. (2016). Effects of the a flavonoid-rich fraction on the acquisition and extinction of fear memory: pharmacological and molecular approaches. *Front. Behav. Neurosci.* 9:345. 10.3389/fnbeh.2015.00345 26778988 PMC4700274

[B65] Oliveira D. R. Sanada P. F. Filho A. C. S. Conceição G. M. S. Cerutti J. M. Cerutti S. M. (2013). Long-term treatment with standardized extract of *Ginkgo biloba* L. enhances the conditioned suppression of licking in rats by the modulation of neuronal and glial cell function in the dorsal hippocampus and central amygdala. *Neuroscience* 235 70–86. 10.1016/j.neuroscience.2013.01.009 23321541

[B66] Oliveira D. R. Sanada P. F. Saragossa F. A. C. Innocenti L. R. Oler G. Cerutti J. M. (2009). Neuromodulatory property of standardized extract *Ginkgo biloba* L. *(EGb* 761) on memory: behavioral and molecular evidence. *Brain Res.* 1269 68–89. 10.1016/j.brainres.2008.11.105 19146837

[B67] Osterweil E. Wells D. G. Mooseker M. S. (2005). A role for myosin VI in postsynaptic structure and glutamate receptor endocytosis. *J. Cell Biol.* 168 329–338. 10.1083/jcb.200410091 15657400 PMC2171578

[B68] Ousman S. S. Frederick A. Lim E. M. F. (2017). Chaperone proteins in the central nervous system and peripheral nervous system after nerve injury. *Front. Neurosci.* 11:79 10.3389/fnins.2017.00079 PMC5318438 28270745

[B69] Pedroso A. P. Watanabe R. L. H. Albuquerque K. T. Telles M. M. Andrade M. C. C. Perez J. D. (2012). Proteomic profiling of the rat hypothalamus. *Proteome Sci.* 10 26–40. 10.1186/1477-5956-10-26 22519962 PMC3441799

[B70] Ribeiro M. L. Moreira L. M. Arçari D. P. dos Santos L. F. Marques A. C. Pedrazzoli J. (2016). Protective effects of chronic treatment with a standardized extract of *Ginkgo biloba* L. in the prefrontal cortex and dorsal hippocampus of middle-aged rats. *Behav. Brain Res.* 313 144–150. 10.1016/j.bbr.2016.06.029 27424157

[B71] Saraiva L. M. Seixas da Silva G. S. Galina A. da-Silva W. S. Klein W. L. Ferreira S. T. (2010). Amyloid-β triggers the release of neuronal Hexokinase 1 from mitochondria. *PLoS One* 5:e15230. 10.1371/journal.pone.0015230 21179577 PMC3002973

[B72] Serrano-García N. Pedraza-Chaverri J. Mares-Sámano J. J. Orozco-Ibarra M. Cruz-Salgado A. Jiménez-Anguiano A. (2013). Antiapoptotic effects of EGb 761. *Evid. Based Complement. Alternat. Med.* 2013:495703. 10.1155/2013/495703 23983787 PMC3745884

[B73] Shevchenko A. Wilm M. Vorm O. Mann M. (1996). Mass spectrometric sequencing of proteins from silver-stained polyacrylamide gels. *Anal. Chem.* 68 850–858. 10.1021/ac950914h 8779443

[B74] Shields L. Y. Kim H. Zhu L. Haddad D. Berthet A. Pathak D. (2015). Dynamin-related protein 1 is required for normal mitochondrial bioenergetic and synaptic function in CA1 hippocampal neurons. *Cell Death Dis.* 6:e1725. 10.1038/cddis.2015.94 25880092 PMC4650558

[B75] Stark M. Behl C. (2014). The *Ginkgo biloba* extract EGb 761 modulates proteasome activity and polyglutamine protein aggregation. *Evid. Based Complement. Altern. Med.* 2014 1–14. 10.1155/2014/940186 25002904 PMC4068065

[B76] Stetler R. A. Gan Y. Zhang W. Liou A. K. Gao Y. Cao G. (2010). Heat shock proteins: cellular and molecular mechanisms in the central nervous system. *Prog. Neurobiol.* 92 184–211. 10.1016/j.pneurobio.2010.05.002 20685377 PMC2939168

[B77] Stork O. (1999). Memory formation and the regulation of gene expression. *Cell Mol. Life Sci.* 55 575–592. 10.1007/s000180050316 10357228 PMC11146995

[B78] Strange R. C. Spiteri M. A. Ramachandran S. Fryer A. A. (2001). Glutathione-S-transferase family of enzymes. *Mutat. Res. Mol. Mech. Mutagen.* 482 21–26. 10.1016/S0027-5107(01)00206-8 11535245

[B79] Subhan Z. Hindmarch I. (1984). The psychopharmacological effects of *Ginkgo biloba* in normal healthy volunteers. *Int. J. Clin. Pharmacol. Res.* 4 89–93. 6469442

[B80] Tan M. S. Yu J. T. Tan C. C. Wang H. F. Meng X. F. Wang C. (2015). Efficacy and adverse effects of *Ginkgo biloba* for cognitive impairment and dementia: a systematic review and meta-analysis. *J. Alzheimer’s Dis.* 43 589–603. 10.3233/JAD-140837 25114079

[B81] Tanaka S. Uehara T. Nomura Y. (2000). Up-regulation of protein-disulfide isomerase in response to hypoxia/brain ischemia and its protective effect against apoptotic cell death. *J. Biol. Chem.* 275 10388–10393. 10.1074/jbc.275.14.10388 10744727

[B82] Tchantchou F. Xu Y. Wu Y. Christen Y. Luo Y. (2007). EGb 761 enhances adult hippocampal neurogenesis and phosphorylation of CREB in transgenic mouse model of Alzheimer’s disease. *FASEB J.* 21 2400–2408. 10.1096/fj.06-7649com 17356006

[B83] Wang Y. Xu Y. Liu Q. Zhang Y. Gao Z. Yin M. (2017). Myosin IIA-related actomyosin contractility mediates oxidative stress-induced neuronal apoptosis. *Front. Mol. Neurosci.* 10:75. 10.3389/fnmol.2017.00075 28352215 PMC5348499

[B84] Woelk H. Arnoldt K. H. Kieser M. Hoerr R. (2007). *Ginkgo biloba* special extract EGb 761 in generalized anxiety disorder and adjustment disorder with anxious mood: a randomized, double-blind, placebo-controlled trial. *J. Psychiatr. Res.* 41 472–480. 10.1016/j.jpsychires.2006.05.004 16808927

[B85] Won J. Silva A. J. (2008). Molecular and cellular mechanisms of memory allocation in neuronetworks. *Neurobiol. Learn. Mem.* 89 285–292. 10.1016/j.nlm.2007.08.017 17962049 PMC2673809

[B86] Wylie S. R. Chantler P. D. (2003). Myosin {IIA} drives neurite retraction. *Mol. Biol. Cell* 14 4654–4666. 10.1091/mbc.e03-03-0187 12960431 PMC266780

[B87] Yau S. Li A. So K.-F. (2015). Involvement of adult hippocampal neurogenesis in learning and forgetting. *Neural Plast.* 2015 1–13. 10.1155/2015/717958 26380120 PMC4561984

[B88] Yoo D. Y. Jung H. Y. Kim J. W. Yim H. S. Kim D. W. Nam H. (2016). Reduction of dynamin 1 in the hippocampus of aged mice is associated with the decline in hippocampal-dependent memory. *Mol. Med. Rep.* 14 4755–4760. 10.3892/mmr.2016.5804 27748822

[B89] Yoshitake T. Yoshitake S. Kehr J. (2010). The *Ginkgo biloba* extract EGb 761(R) and its main constituent flavonoids and ginkgolides increase extracellular dopamine levels in the rat prefrontal cortex. *Br. J. Pharmacol.* 159 659–668. 10.1111/j.1476-5381.2009.00580.x 20105177 PMC2828029

[B90] Yuan Q. Wang C. Shi J. Lin Z. (2017). Effects of *Ginkgo biloba* on dementia: an overview of systematic reviews. *J. Ethnopharmacol.* 195 1–9. 10.1016/j.jep.2016.12.005 27940086

[B91] Zamberlam C. R. Vendrasco N. C. Oliveira D. R. Gaiardo R. B. Cerutti S. M. (2016). Effects of standardized *Ginkgo biloba* extract on the acquisition, retrieval and extinction of conditioned suppression: evidence that short-term memory and long-term memory are differentially modulated. *Physiol. Behav.* 165 55–68. 10.1016/j.physbeh.2016.06.036 27378507

[B92] Zhang H. Kang E. Wang Y. Yang C. Yu H. Wang Q. (2016). Brain-specific Crmp2 deletion leads to neuronal development deficits and behavioural impairments in mice. *Nat. Commun.* 7 1–11. 10.1038/ncomms11773 27249678 PMC4895353

