# Supplementary material for: Target Proteins in the Dorsal Hippocampal Formation Sustain the Memory-Enhancing and Neuroprotective Effects of Ginkgo biloba
Source: Front Pharmacol. 2019 Jan 7;9:1533. doi: 10.3389/fphar.2018.01533 (PMC6330356; doi:10.3389/fphar.2018.01533)
Supplement: Supplementary file 1 [file Table_1.DOCX]

**Supplementary Table 1 –** SR means of conditioned lick suppression during the retention test session.

| Trails | Vehicle | 0.25 g.kg^-1^ EGb | 0.50 g.kg^-1^ EGb | 1.00 g.kg^-1^ EGb |
| --- | --- | --- | --- | --- |
| 1 | 0.89 ± 0.02 | 0.77 ± 0.05 | 0.78 ± 0.05 | 0.82 ± 0.05 |
| 2,3,4 | 0.55 ± 0.04* | 0.55 ± 0.05* | 0.55 ± 0.02* | 0.50 ± 0.05* |
| 5,6,7 | 0.45 ± 0.05* | 0.64 ± 0.06 * | 0.52 ± 0.03* | 0.61 ± 0.07* |
| 8,9,10 | 0.56 ± 0.06* | 0.55 ± 0.07* | 0.54 ± 0.04* | 0.52 ± 0.07* |

Data represent mean ± SEM (n=6 per group). *P<0.05 (two-way ANOVA repeated measures-Tukey post hoc test) when compared with the first trial.
