# Supplementary material for: Target Proteins in the Dorsal Hippocampal Formation Sustain the Memory-Enhancing and Neuroprotective Effects of Ginkgo biloba
Source: Front Pharmacol. 2019 Jan 7;9:1533. doi: 10.3389/fphar.2018.01533 (PMC6330356; doi:10.3389/fphar.2018.01533)
Supplement: Supplementary file 2 [file Table_2.docx]

**Supplementary Table 2** – Optical density means of each protein differentially expressed between the control (vehicle) and EGb-treated groups.

| Accession Number | Vehicle | 0.25 g.kg^-1^ EGb | 0.50 g.kg^-1^ EGb | 1.00 g.kg^-1^ EGb |
| --- | --- | --- | --- | --- |
| O35179 | 100.0 ± 6.3 | 39.5 ± 9.3 | 64.1 ± 13.3 | 46.7 ± 15.9 |
| P00564 | 100.0 ± 22.5 | 73.5 ± 3.8 | 148.3 ± 23.8 | 60.1 ± 22.1 |
| P02563 | 100.0 ± 9.0 | 100.9 ± 12.1 | 140.4 ± 16.9 | 64.1 ± 11.8 |
| P04462 | 100.0 ± 32.8 | 328.2 ± 91.7 | 409.4 ± 62.6 | 170.7 ± 41.2 |
| P05708 | 100.0 ± 27.2 | 107.6 ± 17.9 | 205.6 ± 5.0 | 118.4 ± 21.9 |
| P11598 | 100.0 ± 19.6 | 110.6 ± 24.1 | 209.4 ± 28.2 | 65.7 ± 12.5 |
| P13471 | 100.0 ± 17.9 | 70.6 ± 20.4 | 46.3 ± 10.2 | 159.0 ± 43.3 |
| P18420 | 100.0 ± 13.9 | 26.1 ± 8.9 | 111.3 ± 26.9 | 68.6 ± 21.9 |
| P21575 | 100.0 ± 33.2 | 70.2 ± 9.4 | 188.7 ± 16.3 | 81.7 ± 16.4 |
| P25113 | 100.0 ± 15.4 | 324.1 ± 23.0 | 251.9 ± 54.9 | 232.3 ± 52.9 |
| P27605 | 100.0 ± 47.2 | 339.6 ± 113.1 | 499.3 ± 257.4 | 4198.7 ± 1957.1 |
| P38983 | 100.0 ± 5.9 | 75.1 ± 14.5 | 45.9 ± 15.5 | 51.5 ± 6.8 |
| P47942 | 100.0 ± 27.5 | 204.6 ± 30.4 | 251.9 ± 36.4 | 208.3 ± 12.4 |
| P48004 | 100.0 ± 3.4 | 56.5 ± 7.0 | 43.3 ± 9.4 | 71.5 ± 10.3 |
| P60901 | 100.0 ± 29.6 | 158.6 ± 72.5 | 566.7 ± 58.5 | 676.7 ± 191.1 |
| P68511 | 100.0 ± 24.7 | 76.7 ± 17.6 | 209.0 ± 41.8 | 53.9 ± 8.5 |
| P82995 | 100.0 ± 38.7 | 98.0 ± 32.7 | 238.0 ± 11.2 | 85.8 ± 19.2 |
| P86252 | 100.0 ± 23.0 | 123.8 ± 25.1 | 175.7 ± 23.3 | 81.7 ± 15.8 |
| Q01986 | 100.0 ± 21.9 | 327.2 ± 38.5 | 201.8 ± 52.1 | 209.2 ± 60.2 |
| Q29RW1 | 100.0 ± 8.3 | 88.7 ± 21.9 | 181.3 ± 24.3 | 84.8 ± 25.1 |
| Q3KR86 | 100.0 ± 9.8 | 133.4 ± 21.1 | 215.8 ± 41.5 | 79.7 ± 11.1 |
| Q5RKI0 | 100.0 ± 31.2 | 261.0 ± 53.4 | 230.5 ± 17.0 | 105.2 ± 21.7 |
| Q62950 | 100.0 ± 22.3 | 213.6 ± 30.6 | 171.7 ± 10.5 | 74.5 ± 16.3 |
| Q63537 | 100.0 ± 33.1 | 180.0 ± 27.3 | 87.1 ± 25.6 | 62.2 ± 15.6 |
| Q63754 | 100.0 ± 24.3 | 192.3 ± 40.0 | 279.8 ± 76.0 | 88.4 ± 21.6 |
| Q7TPB1 | 100.0 ± 22.9 | 430.2 ± 68.1 | 244.0 ± 73.0 | 291.0 ± 62.4 |
| Q9JHU0 | 100.0 ± 37.8 | 285.3 ± 14.7 | 156.5 ± 36.9 | 164.2 ± 45.5 |
| Q9QUL6 | 100.0 ± 38.4 | 44.3 ± 16.3 | 181 ± 30.7 | 129 ± 19.2 |
| 5FWG_A | 100.0 ± 51.2 | 284.9 ± 61.6 | 424.2 ± 71.9 | 610.6 ± 119.9 |
| NP_001101931 | 100.0 ± 23.8 | 146.7 ± 72.0 | 366.2 ± 56.9 | 122.4 ± 13.8 |
| NP_001128629 | 100.0 ± 33.8 | 185.0 ± 32.6 | 254.6 ± 24.7 | 104.9 ± 30.7 |
| NP_001166900 | 100.0 ± 14.9 | 125.7 ± 15.6 | 195.0 ± 26.6 | 185.9 ± 8.9 |

Accession number of Swiss-Prot or NCBI-prot databases. Data was expressed as percentage change relative to the basal levels (control) mean ± SEM (n=5 per group).
