# Supplementary material for: Target Proteins in the Dorsal Hippocampal Formation Sustain the Memory-Enhancing and Neuroprotective Effects of Ginkgo biloba
Source: Front Pharmacol. 2019 Jan 7;9:1533. doi: 10.3389/fphar.2018.01533 (PMC6330356; doi:10.3389/fphar.2018.01533)
Supplement: Supplementary file 3 [file Table_3.DOCX]

**Supplementary Table 3 –** Mass spectrometry data of the thirty-two dorsal hippocampal formation proteins significantly affected by EGb-treatment and conditioned lick suppression.

| Accession Number^1^ | Protein Description | Gene Name | MW^2^ | pI | PS^3^ | MP^4^ | SC^5^ | Peptides | IS^6^ |
| --- | --- | --- | --- | --- | --- | --- | --- | --- | --- |
| O35179 | Endophilin-A1 | Sh3gl2 | 40045 | 5,26 | 74 | 7(3) | 20,2 | K.IPDEELR.Q | 37 |
|  |  |  |  |  |  |  |  | K.LDDDFKEMER.K + Oxidation (M) | 28 |
|  |  |  |  |  |  |  |  | K.QAVQILQQVTVR.L | 5 |
|  |  |  |  |  |  |  |  | K.TIEYLQPNPASR.A | 19 |
|  |  |  |  |  |  |  |  | K.TIEYLQPNPASR.A | 43 |
|  |  |  |  |  |  |  |  | R.ELSEVKDSLDMEVK.Q + Oxidation (M) | 3 |
|  |  |  |  |  |  |  |  | R.ALYDFEPENEGELGFK.E | 12 |
| P00564 | Creatine kinase M-type | Ckm | 43246 | 6,58 | 178 | 5(3) | 17,3 | K.LSVEALNSLTGEFK.G | 104 |
|  |  |  |  |  |  |  |  | K.SFLVWVNEEDHLR.V | 20 |
|  |  |  |  |  |  |  |  | R.LGSSEVEQVQLVVDGVK.L | 28 |
|  |  |  |  |  |  |  |  | R.GTGGVDTAAVGAVFDISNADR.L | 80 |
|  |  |  |  |  |  |  |  | K.RGTGGVDTAAVGAVFDISNADR.L | 2 |
| P02563 | Myosin-6 | Myh6 | 224168 | 5,59 | 150 | 11(4) | 6,4 | R.SLSTELFK.L | 16 |
|  |  |  |  |  |  |  |  | R.LDEAEQIALK.G | 50 |
|  |  |  |  |  |  |  |  | K.NALAHALQSAR.H | 18 |
|  |  |  |  |  |  |  |  | R.TKYETDAIQR.T | 1 |
|  |  |  |  |  |  |  |  | K.DIDDLELTLAK.V | 11 |
|  |  |  |  |  |  |  |  | R.IEELEEELEAER.T | 3 |
|  |  |  |  |  |  |  |  | R.IEELEEELEAER.T | 60 |
|  |  |  |  |  |  |  |  | K.AITDAAMMAEELKK.E | 3 |
|  |  |  |  |  |  |  |  | R.LQNEIEDLMVDVER.S | 23 |
|  |  |  |  |  |  |  |  | R.DLEEATLQHEATAAALR.K | 58 |
|  |  |  |  |  |  |  |  | K.HADSVAELGEQIDNLQR.V | 37 |
| P04462 | Myosin-8 | Myh8 | 223698 | 5,63 | 398 | 24(8) | 14,2 | K.MFLWMVTR.I | 10 |
|  |  |  |  |  |  |  |  | R.IQLELNQVK.S | 25 |
|  |  |  |  |  |  |  |  | R.LDEAEQLALK.G | 50 |
|  |  |  |  |  |  |  |  | R.TLEDQVSELK.S | 17 |
|  |  |  |  |  |  |  |  | R.TKYETDAIQR.T | 1 |
|  |  |  |  |  |  |  |  | K.DIDDLELTLAK.V | 11 |
|  |  |  |  |  |  |  |  | K.DTQLHLDDALR.G | 17 |
|  |  |  |  |  |  |  |  | K.GSSFQTVSALFR.E | 80 |
|  |  |  |  |  |  |  |  | K.LTGAVMHYGNMK.F | 8 |
|  |  |  |  |  |  |  |  | K.LTGAVMHYGNMK.F + Oxidation (M) | 4 |
|  |  |  |  |  |  |  |  | R.ANLLQAEIEELR.A | 4 |
|  |  |  |  |  |  |  |  | R.IEELEEEIEAER.A | 3 |
|  |  |  |  |  |  |  |  | R.IEELEEEIEAER.A | 60 |
|  |  |  |  |  |  |  |  | K.AITDAAMMAEELKK.E | 3 |
|  |  |  |  |  |  |  |  | K.VLNASAIPEGQFIDSK.K | 19 |
|  |  |  |  |  |  |  |  | R.LQNEVEDLMLDVER.T | 57 |
|  |  |  |  |  |  |  |  | R.VQLLHTQNTSLINTK.K | 66 |
|  |  |  |  |  |  |  |  | K.TPGAMEHELVLHQLR.C | 37 |
|  |  |  |  |  |  |  |  | K.TPGAMEHELVLHQLR.C + Oxidation (M) | 1 |
|  |  |  |  |  |  |  |  | K.NLQQEISDLTEQIAEGGK.H | 86 |
|  |  |  |  |  |  |  |  | K.LAQESTMDIENDKQQLDEK.L | 18 |
|  |  |  |  |  |  |  |  | K.MQGTLEDQIISANPLLEAFGNAK.T | 87 |
|  |  |  |  |  |  |  |  | K.ALQEAHQQTLDDLQAEEDKVNTLTK.A | 4 |
| P05708 | Hexokinase 1 | Hk1 | 103540 | 6,29 | 406 | 25(16) | 24,6 | R.LILVK.M | 27 |
|  |  |  |  |  |  |  |  | K.GFLFR.G | 12 |
|  |  |  |  |  |  |  |  | R.LALLQVR.A | 17 |
|  |  |  |  |  |  |  |  | K.MLPSFVR.S + Oxidation (M) | 15 |
|  |  |  |  |  |  |  |  | K.MLPTFVR.S + Oxidation (M) | 12 |
|  |  |  |  |  |  |  |  | K.EGLLFEGR.I | 52 |
|  |  |  |  |  |  |  |  | R.ITPELLTR.G | 34 |
|  |  |  |  |  |  |  |  | R.NILIDFTK.K | 41 |
|  |  |  |  |  |  |  |  | R.DYNPTASVK.M | 2 |
|  |  |  |  |  |  |  |  | K.GAALITAVGVR.L | 31 |
|  |  |  |  |  |  |  |  | R.FLLSESGTGK.G | 28 |
|  |  |  |  |  |  |  |  | K.FLSQIESDR.L | 47 |
|  |  |  |  |  |  |  |  | K.GAAMVTAVAYR.L + Oxidation (M) | 58 |
|  |  |  |  |  |  |  |  | R.TTVGVDGSLYK.M | 56 |
|  |  |  |  |  |  |  |  | K.FNTSDVSAIEK.D | 9 |
|  |  |  |  |  |  |  |  | K.FNTSDVSAIEK.D | 54 |
|  |  |  |  |  |  |  |  | K.VVDEYSLNSGK.Q | 80 |
|  |  |  |  |  |  |  |  | R.QIEETLAHFR.L | 4 |
|  |  |  |  |  |  |  |  | K.IDEAVLITWTK.R | 16 |
|  |  |  |  |  |  |  |  | K.MISGMYLGEIVR.N + 2 Oxidation (M) | 31 |
|  |  |  |  |  |  |  |  | K.MVSGMYMGELVR.L + 3 Oxidation (M) | 57 |
|  |  |  |  |  |  |  |  | K.GDFIALDLGGSSFR.I | 40 |
|  |  |  |  |  |  |  |  | R.AAQLCGAGMAAVVEK.I + Oxidation (M) | 40 |
|  |  |  |  |  |  |  |  | K.ATDCEGHDVASLLRD | 14 |
|  |  |  |  |  |  |  |  | K.ATDCEGHDVASLLR.D | 49 |
| P11598 | Protein disulfide-isomerase A3 | Pdia3 | 57044 | 5,88 | 98 | 9(2) | 19,8 | K.LNFAVASR.K | 7 |
|  |  |  |  |  |  |  |  | K.QAGPASVPLR.T | 18 |
|  |  |  |  |  |  |  |  | K.YGVSGYPTLK.I | 34 |
|  |  |  |  |  |  |  |  | K.FVMQEEFSR.D + Oxidation (M) | 60 |
|  |  |  |  |  |  |  |  | R.LAPEYEAAATR.L | 25 |
|  |  |  |  |  |  |  |  | R.DGEEAGAYDGPR.T | 13 |
|  |  |  |  |  |  |  |  | K.GFPTIYFSPANK.K | 5 |
|  |  |  |  |  |  |  |  | K.SEPIPETNEGPVK.V | 20 |
|  |  |  |  |  |  |  |  | K.MDATANDVPSPYEVK.G + Oxidation (M) | 6 |
| P13471 | 40S ribosomal protein S14 | Rps14 | 16420 | 10,07 | 51 | 1(1) | 8,6 | R.IEDVTPIPSDSTR.R | 51 |
| P18420 | Proteasome subunit alpha type-1 | Psma1 | 29784 | 6,15 | 179 | 6(3) | 24 | R.LVSLIGSK.T | 54 |
|  |  |  |  |  |  |  |  | K.NVSIGIVGK.D | 29 |
|  |  |  |  |  |  |  |  | R.LLCNFMR.Q + Oxidation (M) | 6 |
|  |  |  |  |  |  |  |  | R.ETLPAEQDLTTK.N | 28 |
|  |  |  |  |  |  |  |  | R.IHQIEYAMEAVK.Q + Oxidation (M) | 49 |
|  |  |  |  |  |  |  |  | R.NQYDNDVTVWSPQGR.I | 13 |
| P21575 | Dynamin-1 | Dnm1 | 97576 | 6,44 | 168 | 20(6) | 21,4 | R.DFLPR.G | 13 |
|  |  |  |  |  |  |  |  | R.DTLPGLR.N | 19 |
|  |  |  |  |  |  |  |  | R.EISYAIK.N | 27 |
|  |  |  |  |  |  |  |  | R.GYIGVVNR.S | 24 |
|  |  |  |  |  |  |  |  | R.FPFELVK.M | 20 |
|  |  |  |  |  |  |  |  | K.DITAALAAER.K | 56 |
|  |  |  |  |  |  |  |  | K.FTDFEEVR.L | 13 |
|  |  |  |  |  |  |  |  | K.GISPVPINLR.V | 46 |
|  |  |  |  |  |  |  |  | K.YMLSVDNLK.L + Oxidation (M) | 27 |
|  |  |  |  |  |  |  |  | K.SSVLENFVGR.D | 11 |
|  |  |  |  |  |  |  |  | R.DMLMQFVTK.E + 2 Oxidation (M) | 5 |
|  |  |  |  |  |  |  |  | K.FFLSHPSYR.H | 6 |
|  |  |  |  |  |  |  |  | K.KDITAALAAER.K | 10 |
|  |  |  |  |  |  |  |  | K.LQSQLLSIEK.E | 10 |
|  |  |  |  |  |  |  |  | R.GMEDLIPLVNR.L + Oxidation (M) | 13 |
|  |  |  |  |  |  |  |  | K.VLNQQLTNHIR.D | 11 |
|  |  |  |  |  |  |  |  | K.TSGNQDEILVIR.K | 52 |
|  |  |  |  |  |  |  |  | K.TIMHLMINNTK.E + 2 Oxidation (M) | 4 |
|  |  |  |  |  |  |  |  | R.NLVDSYMAIVNK.T + Oxidation (M) | 46 |
|  |  |  |  |  |  |  |  | R.IEGSGDQIDTYELSGGAR.I | 7 |
| P25113 | Phosphoglycerate mutase 1 | Pgam1 | 28928 | 6,67 | 111 | 6(3) | 20,9 | R.HYGGLTGLNK.A | 7 |
|  |  |  |  |  |  |  |  | R.HYGGLTGLNK.A | 34 |
|  |  |  |  |  |  |  |  | R.VLIAAHGNSLR.G | 34 |
|  |  |  |  |  |  |  |  | R.VLIAAHGNSLR.G | 5 |
|  |  |  |  |  |  |  |  | R.HGESAWNLENR.F | 83 |
|  |  |  |  |  |  |  |  | R.SYDVPPPPMEPDHPFYSNISK.D + Oxidation (M) | 3 |
| P27605 | Hypoxanthine-guanine phosphoribosyltransferase | Hprt1 | 24690 | 6,07 | 116 | 5(3) | 21,1 | K.TMQTLLSLVK.Q + Oxidation (M) | 30 |
|  |  |  |  |  |  |  |  | R.SIPMTVDFIR.L + Oxidation (M) | 50 |
|  |  |  |  |  |  |  |  | K.VIGGDDLSTLTGK.N | 10 |
|  |  |  |  |  |  |  |  | K.VIGGDDLSTLTGK.N | 88 |
|  |  |  |  |  |  |  |  | K.NVLIVEDIIDTGK.T | 15 |
| P38983 | 40S ribosomal protein SA | Rpsa | 32917 | 4,8 | 63 | 2(1) | 7,1 | R.LLVVTDPR.A | 0 |
|  |  |  |  |  |  |  |  | K.FAAATGATPIAGR.F | 63 |
| P47942 | Dihydropyrimidinase-related protein 2 | Dpysl2 | 62638 | 5,95 | 142 | 6(3) | 14 | K.SAAEVIAQAR.K | 41 |
|  |  |  |  |  |  |  |  | R.GSPLVVISQGK.I | 39 |
|  |  |  |  |  |  |  |  | K.GIQEEMEALVK.D + Oxidation (M) | 22 |
|  |  |  |  |  |  |  |  | K.MDENQFVAVTSTNAAK.V + Oxidation (M) | 79 |
|  |  |  |  |  |  |  |  | K.DNFTLIPEGTNGTEER.M | 17 |
|  |  |  |  |  |  |  |  | R.SITIANQTNCPLYVTK.V | 24 |
| P48004 | Proteasome subunit alpha type-7 | Psma7 | 28010 | 8,6 | 141 | 5(3) | 23,4 | R.DIVVLGVEK.K | 23 |
|  |  |  |  |  |  |  |  | K.ILSPEEIEK.Y | 32 |
|  |  |  |  |  |  |  |  | K.ALLEVVQSGGK.N | 46 |
|  |  |  |  |  |  |  |  | R.LTVEDPVTVEYITR.Y | 11 |
|  |  |  |  |  |  |  |  | K.NYTDDAIETDDLTIK.L | 88 |
| P60901 | Proteasome subunit alpha type-6 | Psma6 | 27838 | 6,34 | 196 | 7(5) | 33,7 | R.HITIFSPEGR.L | 47 |
|  |  |  |  |  |  |  |  | R.LYQVEYAFK.A | 55 |
|  |  |  |  |  |  |  |  | K.QTESTSFLEK.K | 53 |
|  |  |  |  |  |  |  |  | K.AINQGGLTSVAVR.G | 70 |
|  |  |  |  |  |  |  |  | K.LLDSSTVTHLFK.I | 43 |
|  |  |  |  |  |  |  |  | K.YGYEIPVDMLCK.R + Oxidation (M) | 15 |
|  |  |  |  |  |  |  |  | K.ITENIGCVMTGMTADSR.S + 2 Oxidation (M) | 18 |
| P68511 | 14-3-3 protein eta | Ywhah | 28365 | 4,81 | 348 | 9(7) | 18,3 | R.NLLSVAYK.N | 9 |
|  |  |  |  |  |  |  |  | R.YLAEVASGEK.K | 68 |
|  |  |  |  |  |  |  |  | R.YLAEVASGEKK.N | 6 |
|  |  |  |  |  |  |  |  | K.NSVVEASEAAYK.E | 52 |
|  |  |  |  |  |  |  |  | K.NSVVEASEAAYK.E | 44 |
|  |  |  |  |  |  |  |  | K.KNSVVEASEAAYK.E | 53 |
|  |  |  |  |  |  |  |  | K.KNSVVEASEAAYK.E | 88 |
|  |  |  |  |  |  |  |  | K.AVTELNEPLSNEDR.N | 58 |
|  |  |  |  |  |  |  |  | K.AVTELNEPLSNEDR.N | 89 |
| P82995 | Heat shock protein HSP 90-alpha | Hsp90aa1 | 85161 | 4,93 | 149 | 7(3) | 10,5 | K.FYEQFSK.N | 4 |
|  |  |  |  |  |  |  |  | K.YIDQEELNK.T | 16 |
|  |  |  |  |  |  |  |  | K.DQVANSAFVER.L | 38 |
|  |  |  |  |  |  |  |  | R.ELISNSSDALDK.I | 7 |
|  |  |  |  |  |  |  |  | K.EDQTEYLEER.R | 48 |
|  |  |  |  |  |  |  |  | R.GVVDSEDLPLNISR.E | 29 |
|  |  |  |  |  |  |  |  | R.YYTSASGDEMVSLK.D + Oxidation (M) | 5 |
| P86252 | Transcriptional activator protein Pur-alpha | Pura | 35157 | 6,07 | 88 | 6(4) | 19,1 | K.SEFLVR.E | 21 |
|  |  |  |  |  |  |  |  | R.FYLDVK.Q | 37 |
|  |  |  |  |  |  |  |  | K.YGVFMR.V + Oxidation (M) | 28 |
|  |  |  |  |  |  |  |  | R.NSITVPYK.V | 40 |
|  |  |  |  |  |  |  |  | R.FFFDVGSNK.Y | 50 |
|  |  |  |  |  |  |  |  | K.LIDDYGVEEEPAELPEGTSLTVDNKR.F | 3 |
| Q01986 | Dual specificity mitogen-activated protein kinase kinase 1 | Map2k1 | 43779 | 6,18 | 83 | 6(4) | 12,7 | K.GLTYLR.E | 11 |
|  |  |  |  |  |  |  |  | K.VSIAVIK.G | 47 |
|  |  |  |  |  |  |  |  | R.IPEQILGK.V | 22 |
|  |  |  |  |  |  |  |  | R.LEAFLTQK.Q | 22 |
|  |  |  |  |  |  |  |  | R.YPIPPPDAK.E | 28 |
|  |  |  |  |  |  |  |  | R.DVKPSNILVNSR.G | 34 |
| Q29RW1 | Myosin-4 | Myh4 | 223653 | 5,58 | 320 | 8(7) | 4,1 | R.IEELEEEIEAER.A | 68 |
|  |  |  |  |  |  |  |  | R.VIQYFATIAVTGDK.K | 25 |
|  |  |  |  |  |  |  |  | R.AEDEEEINAELTAK.K | 51 |
|  |  |  |  |  |  |  |  | R.VVESMQSTLDAEIR.S | 96 |
|  |  |  |  |  |  |  |  | R.VVESMQSTLDAEIR.S + Oxidation (M) | 34 |
|  |  |  |  |  |  |  |  | R.VVESMQSTLDAEIR.S + Oxidation (M) | 49 |
|  |  |  |  |  |  |  |  | K.TLAFLFSGGQAAEAEGGGGK.K | 64 |
|  |  |  |  |  |  |  |  | K.ELTERAEDEEEINAELTAK.K | 57 |
| Q3KR86 | MICOS complex subunit Mic60 | Immt | 67477 | 5,57 | 75 | 5(1) | 10,5 | R.GVYSEETLR.A | 2 |
|  |  |  |  |  |  |  |  | R.KAVDEAADALLK.A | 6 |
|  |  |  |  |  |  |  |  | R.QTITAQNAAVQAVK.A | 4 |
|  |  |  |  |  |  |  |  | K.SLEDALNQTATVTR.Q | 45 |
|  |  |  |  |  |  |  |  | R.EIAGATPYITAAEEK.L | 17 |
| Q5RKI0 | WD repeat-containing protein 1 | Wdr1 | 66824 | 6,15 | 452 | 11(8) | 18,3 | K.EWTITY | 27 |
|  |  |  |  |  |  |  |  | R.YTNLTLR.D | 44 |
|  |  |  |  |  |  |  |  | K.VINSVDIK.Q | 65 |
|  |  |  |  |  |  |  |  | R.VYSILGATLK.D | 44 |
|  |  |  |  |  |  |  |  | K.SIQCLTVHK.N | 18 |
|  |  |  |  |  |  |  |  | K.YEYQPFAGK.I | 21 |
|  |  |  |  |  |  |  |  | K.VFASLPQVER.G | 1 |
|  |  |  |  |  |  |  |  | K.DIAWTEDSKR.I | 41 |
|  |  |  |  |  |  |  |  | R.VYSILGATLKDEGK.L | 62 |
|  |  |  |  |  |  |  |  | K.YAPSGFYIASGDISGK.L | 101 |
|  |  |  |  |  |  |  |  | R.NIDNPAVADIYTEHAHQVVVAK.Y | 28 |
| Q62950 | Dihydropyrimidinase-related protein 1 | Crmp1 | 62499 | 6,64 | 99 | 9(4) | 14,2 | K.IFNLYPR.K | 26 |
|  |  |  |  |  |  |  |  | K.SAADIIALAR.K | 48 |
|  |  |  |  |  |  |  |  | R.INCPVYITK.V | 7 |
|  |  |  |  |  |  |  |  | K.IVFEDGNISVSK.G | 8 |
|  |  |  |  |  |  |  |  | K.GVNSFQVYMAYK.D + Oxidation (M) | 7 |
|  |  |  |  |  |  |  |  | R.GMYDGPVYEVPATPK.H | 3 |
|  |  |  |  |  |  |  |  | R.GMYDGPVYEVPATPK.H + Oxidation (M) | 13 |
|  |  |  |  |  |  |  |  | R.GMYDGPVYEVPATPK.H + Oxidation (M) | 56 |
|  |  |  |  |  |  |  |  | K.MDENQFVAVTSTNAAK.I + Oxidation (M) | 25 |
| Q63537 | Synapsin-2 | Syn2 | 63702 | 8,73 | 80 | 3(3) | 5,5 | K.SFASLFSD | 24 |
|  |  |  |  |  |  |  |  | K.ILGDYDIK.V | 27 |
|  |  |  |  |  |  |  |  | K.TNTGSAMLEQIAMSDR.Y + 2 Oxidation (M) | 61 |
| Q63754 | Beta-synuclein | Sncb | 14495 | 4,48 | 126 | 3(2) | 17,5 | K.EGVLYVGSK.T | 22 |
|  |  |  |  |  |  |  |  | K.TKEGVLYVGSKT. | 51 |
|  |  |  |  |  |  |  |  | K.EGVVQGVASVAEK.T | 54 |
| Q7TPB1 | T-complex protein 1 subunit delta | Cct4 | 58576 | 8,24 | 56 | 4(2) | 8,9 | K.LVIEEAER.S | 24 |
|  |  |  |  |  |  |  |  | R.TLSGMESYCVR.A + Oxidation (M) | 27 |
|  |  |  |  |  |  |  |  | K.VIDPATATSVDLR.D | 34 |
|  |  |  |  |  |  |  |  | R.ALIAGGGAPEIELALR.L | 7 |
| Q9JHU0 | Dihydropyrimidinase-related protein 5 | Dpysl5 | 62071 | 6,6 | 112 | 9(6) | 15,8 | R.DFGAIPR.V | 10 |
|  |  |  |  |  |  |  |  | R.DFGAIPR.V | 32 |
|  |  |  |  |  |  |  |  | K.DLYMLR.D + Oxidation (M) | 31 |
|  |  |  |  |  |  |  |  | K.ILNLYPR.K | 39 |
|  |  |  |  |  |  |  |  | R.SFPDIVYK.K | 12 |
|  |  |  |  |  |  |  |  | K.GVNSFQMFMTYK.D + 2 Oxidation (M) | 26 |
|  |  |  |  |  |  |  |  | R.TPYLGDVAVVVNPGK.K | 55 |
|  |  |  |  |  |  |  |  | R.VVYENGVFMCAEGTGK.F + Oxidation (M) | 28 |
|  |  |  |  |  |  |  |  | R.IIPGADADVVVWDPEATK.T | 6 |
| Q9QUL6 | Vesicle-fusing ATPase | Nsf | 83170 | 6,55 | 89 | 8(3) | 11,8 | K.VVNGPEILNK.Y | 5 |
|  |  |  |  |  |  |  |  | K.YVGESEANIR.K | 55 |
|  |  |  |  |  |  |  |  | R.LLDYVPIGPR.F | 33 |
|  |  |  |  |  |  |  |  | K.AENSSLNLIGK.A | 21 |
|  |  |  |  |  |  |  |  | K.GILLYGPPGCGK.T | 23 |
|  |  |  |  |  |  |  |  | K.DIEAMDPSILK.G + Oxidation (M) | 23 |
|  |  |  |  |  |  |  |  | K.IAEESNFPFIK.I | 27 |
|  |  |  |  |  |  |  |  | R.VLDDGELLVQQTK.N | 4 |
| 5FWG_A | Chain A, Tetra-(5-Fluorotryptophanyl)-Glutathione Transferase | - | 25637 | 8,42 | 53 | 4(2) | 17,1 | K.LAQASNK | 7 |
|  |  |  |  |  |  |  |  | K.LYSEFLGK.R | 44 |
|  |  |  |  |  |  |  |  | K.CLDAFPNLK.D | 3 |
|  |  |  |  |  |  |  |  | R.LLLEYTDSSYEEK.R | 28 |
| NP_001101931 | Tubulin polymerization-promoting protein | Tppp | 23703 | 9,45 | 73 | 1(1) | 6,4 | R.VDLVDESGYVPGYK.H | 73 |
| NP_001128629 | Myosin heavy chain IIa | Myh2 | 224152 | 5,61 | 429 | 27(10) | 15,9 | R.ILYADFK.Q | 13 |
|  |  |  |  |  |  |  |  | K.MFLWMVTR.I | 10 |
|  |  |  |  |  |  |  |  | R.IQLELNQVK.S | 25 |
|  |  |  |  |  |  |  |  | R.LDEAEQLALK.G | 50 |
|  |  |  |  |  |  |  |  | R.TLEDQVSELK.S | 17 |
|  |  |  |  |  |  |  |  | R.TKYETDAIQR.T | 1 |
|  |  |  |  |  |  |  |  | K.DIDDLELTLAK.V | 11 |
|  |  |  |  |  |  |  |  | K.DTQLHLDDALR.G | 17 |
|  |  |  |  |  |  |  |  | K.GSSFQTVSALFR.E | 80 |
|  |  |  |  |  |  |  |  | K.LTGAVMHYGNMK.F | 8 |
|  |  |  |  |  |  |  |  | K.LTGAVMHYGNMK.F + Oxidation (M) | 4 |
|  |  |  |  |  |  |  |  | R.ANLLQAEIEELR.A | 4 |
|  |  |  |  |  |  |  |  | R.IEELEEEIEAER.A | 3 |
|  |  |  |  |  |  |  |  | R.IEELEEEIEAER.A | 60 |
|  |  |  |  |  |  |  |  | K.QAFTQQIEELKR.Q | 11 |
|  |  |  |  |  |  |  |  | K.AITDAAMMAEELKK.E | 3 |
|  |  |  |  |  |  |  |  | R.LEEAGGATSAQIEMNK.K | 4 |
|  |  |  |  |  |  |  |  | R.LEEAGGATSAQIEMNK.K + Oxidation (M) | 12 |
|  |  |  |  |  |  |  |  | R.LQNEVEDLMLDVER.T | 57 |
|  |  |  |  |  |  |  |  | R.VQLLHTQNTSLINTK.K | 66 |
|  |  |  |  |  |  |  |  | K.TPGAMEHELVLHQLR.C | 37 |
|  |  |  |  |  |  |  |  | K.TPGAMEHELVLHQLR.C + Oxidation (M) | 1 |
|  |  |  |  |  |  |  |  | K.NAYEESLDQLETLKR.E | 31 |
|  |  |  |  |  |  |  |  | K.HADSVAELGEQIDNLQR.V | 37 |
|  |  |  |  |  |  |  |  | K.NLQQEISDLTEQIAEGGK.R | 86 |
|  |  |  |  |  |  |  |  | K.MQGTLEDQIISANPLLEAFGNAK.T | 87 |
|  |  |  |  |  |  |  |  | K.ALQEAHQQTLDDLQAEEDKVNTLTK.A | 4 |
| NP_001166900 | Septin-6 | Sept6 | 49417 | 6,23 | 130 | 6(4) | 15,9 | K.SLDLVTMK.K + Oxidation (M) | 43 |
|  |  |  |  |  |  |  |  | K.VNIIPIIAK.S | 42 |
|  |  |  |  |  |  |  |  | K.RNEFLGELQK.K | 8 |
|  |  |  |  |  |  |  |  | K.STLMDTLFNTK.F + Oxidation (M) | 19 |
|  |  |  |  |  |  |  |  | R.SSTYDLQESNVGLK.L | 52 |
|  |  |  |  |  |  |  |  | K.FEGEPTTHTQPGVQLR.S | 42 |

^1^Accession number of Swiss-Prot or NCBI-prot databases.

^2^Molecular Weight express as Dalton.

^3^Protein score: protein overall scores higher than 25 are significant (P<0.05).

^4^Matched peptides. Numbers in parenthesis indicate the significant matched peptides.

^5^Sequence coverage.

^6^Ion score
